# The cGAS–STING pathway in tumor immunity: dual roles, regulatory mechanisms, and precision therapeutic strategies

**DOI:** 10.3389/fphar.2026.1844637

**Published:** 2026-07-08

**Authors:** Haiyan Jiang, Zhanzhan Li, Xia Li, Lulu Yu, Jiacheng Sun, Yuntian Shen, Hualin Sun, Lei Qi

**Affiliations:** 1 Department of Emergency Medicine, Affiliated Hospital of Nantong University, Medical School of Nantong University, Nantong University, Nantong, China; 2 Jiangsu Key Laboratory of Tissue Engineering and Neuroregeneration, Key Laboratory of Neuroregeneration of Ministry of Education, Co-innovation Center of Neuroregeneration, Medical School of Nantong University, Nantong University, Nantong, China

**Keywords:** cyclic GMP-AMP synthase–stimulator of interferon genes pathway, combination therapy, innate immunity, STING agonists, tumor immunotherapy

## Abstract

The cyclic GMP-AMP synthase (cGAS)–stimulator of interferon genes (STING) pathway, the core DNA-sensing mechanism in innate immunity, plays a pivotal role in linking tumorigenesis and the immune response. This review systematically elucidates the molecular activation mechanisms of this pathway and its complex regulatory networks within tumors, with a particular focus on analyzing its dual functions—tumor immune surveillance versus tumor promotion—and the determining factors involved. Current research indicates that acute activation of the cGAS–STING pathway potently suppresses tumors by inducing type I interferon responses, thereby promoting dendritic cell (DC) maturation and cytotoxic T lymphocyte infiltration. However, its chronic, persistent activation can paradoxically accelerate tumor progression and immune evasion by remodeling the immunosuppressive tumor microenvironment (TME), inducing chronic inflammation, and enhancing the intrinsic malignant phenotypes of tumor cells. Tumor cells tightly regulate the activity of this pathway through multiple mechanisms, including epigenetic silencing, aberrant post-translational modifications, autophagy-dependent degradation, and tumor microenvironment remodeling. Based on these findings, this review comprehensively summarizes therapeutic strategies targeting the cGAS–STING pathway. These include the latest advancements in STING agonist development, optimization strategies for combination therapies, and innovative applications of nano-delivery systems. Furthermore, we delve into the critical challenges hindering current clinical translation, including pharmacokinetic limitations, mechanisms of tumor resistance, STING genetic polymorphisms, and safety concerns. Finally, we explore future research directions, encompassing precision modulation strategies, personalized therapeutic approaches, and novel delivery systems, aiming to provide a theoretical foundation and innovative insights for the next generation of cancer immunotherapies centered on the cGAS–STING pathway.

## 1 Introduction

Tumor immunotherapy, particularly the clinical application of immune checkpoint inhibitors, has profoundly reshaped the therapeutic landscape for multiple malignant tumors over the past decade, significantly improving patient outcomes (Samson and Ablasser, 2022). However, the prevalence of primary and acquired resistance means that the majority of patients still struggle to achieve durable benefits from existing immunotherapies, urgently necessitating a deeper dissection of the complex mechanisms underlying tumor immune evasion and, consequently, the exploration of innovative therapeutic strategies built upon this understanding (Jiang et al., 2020). It is this pressing need to overcome current efficacy bottlenecks that propels us to expand our research focus from adaptive immunity to the more fundamental innate immune system.

As the body’s first line of defense against microbial invasion, the innate immune system is increasingly recognized for its central role in tumor immunosurveillance (Kwon and Bakhoum, 2020). The discovery of the cyclic GMP-AMP synthase (cGAS)–stimulator of interferon genes (STING) pathway revolutionized our understanding of cytosolic DNA sensing mechanisms in the initiation of innate immunity (Jiang et al., 2026; Gong et al., 2022; Lu et al., 2024a). This pathway not only recognizes pathogenic microbial DNA to launch antiviral defenses but also senses DNA released into the cytosol from the inherent genomic instability or damage of tumor cells themselves, establishing itself as a key molecular node connecting tumorigenesis with the immune response (Zheng et al., 2020; Shen et al., 2025). The elucidation of this mechanism has provided a novel perspective for understanding the intricate interactions between tumors and the immune system. However, as research progresses, it has become increasingly apparent that the role of the cGAS–STING pathway in cancer is not static, with its functional complexity gradually coming to light.

Notably, the role of the cGAS–STING pathway in cancer is highly context-dependent and functionally dualistic. On one hand, a substantial body of research has demonstrated that acute activation of this pathway can effectively initiate anti-tumor immune surveillance. This occurs through the induction of type I interferons, which promote dendritic cell (DC) maturation and antigen cross-presentation, potently activating tumor-specific CD8^+^ T cell-mediated cytotoxicity (Wang et al., 2020). On the other hand, recent studies have revealed that during advanced stages of tumor progression, chronic and sustained activation of the cGAS–STING pathway may instead accelerate tumor growth, metastasis, and immune evasion. This pro-tumor effect is mediated by reshaping the tumor microenvironment (TME), for instance, by fostering inflammation and recruiting immunosuppressive cells such as myeloid-derived suppressor cells (MDSCs) (Kwon and Bakhoum, 2020; Shen et al., 2025). This “double-edged sword” nature necessitates that therapeutic strategies targeting the cGAS–STING pathway be finely tuned in both space and time. The goal is to maximize its anti-tumor efficacy while carefully avoiding potential pro-tumor risks. Recognizing this complexity, researchers are now actively exploring methods to precisely manipulate this pathway to translate these insights into effective clinical treatments.

Given the pathway’s central role in tumor biology and its dualistic functions, therapeutic strategies targeting the cGAS–STING axis are advancing at an unprecedented pace. The development of various STING agonists has progressed to preclinical and clinical study stages, demonstrating promising potential (Zhou et al., 2023). Furthermore, to overcome the inherent limitations of small-molecule agonists, innovative approaches are being explored. These include nanotechnology-based drug delivery systems and combination therapies that pair STING agonists with chemotherapy, radiotherapy, or immune checkpoint inhibitors. Such strategies offer new avenues to surmount current therapeutic bottlenecks and achieve synergistic effects (Wang et al., 2020; Lu et al., 2026). However, in this cutting-edge field, several critical scientific questions remain unresolved: How can the delicate balance between the pathway’s anti-tumor and pro-tumor effects be precisely managed? How can dosing strategies (e.g., dosage, route, and timing) be optimized to enhance efficacy while minimizing systemic toxicity? How can we identify the patient populations most likely to benefit from such therapies (Lu et al., 2026)? In-depth exploration of these questions will ultimately determine whether targeting the cGAS–STING pathway can successfully transition from the laboratory to clinical practice, thereby truly benefiting patients with cancer.

Herein, this review begins with the molecular mechanisms of the cGAS–STING pathway. It systematically discusses its dual role in tumor development and progression, examines the current challenges in targeting this pathway, and comprehensively summarizes the latest research on STING agonists, combination therapy strategies, and novel nano-delivery systems. Finally, we provide a perspective on future directions in this field. Through this systematic synthesis, we aim to establish a robust theoretical foundation and inspire innovative research ideas for the next generation of cancer immunotherapies based on the cGAS–STING pathway.

## 2 Molecular mechanisms of the cGAS–STING pathway

### 2.1 Structure and function of cGAS

cGAS belongs to the nucleotidyltransferase family and is a protein with a molecular weight of approximately 60 kDa. Its structure primarily consists of an N-terminal domain, whose function is not yet fully elucidated, and a highly conserved C-terminal catalytic domain (Shim et al., 2025). The C-terminal domain is central to cGAS function and contains two key structural features: i) a highly conserved catalytic pocket responsible for binding and cyclizing ATP and GTP to generate the second messenger; and ii) a DNA-binding domain capable of recognizing and binding double-stranded DNA (dsDNA) through two independent binding sites. This unique structural architecture enables cGAS to recognize dsDNA longer than 45 base pairs in a sequence-independent manner, ultimately leading to the formation of a stoichiometric cGAS–DNA complex, a critical step for its activation (Shen et al., 2025; Shim et al., 2025). Following this structural overview, we next explore its intracellular localization and activation mechanisms.

**FIGURE 1:**
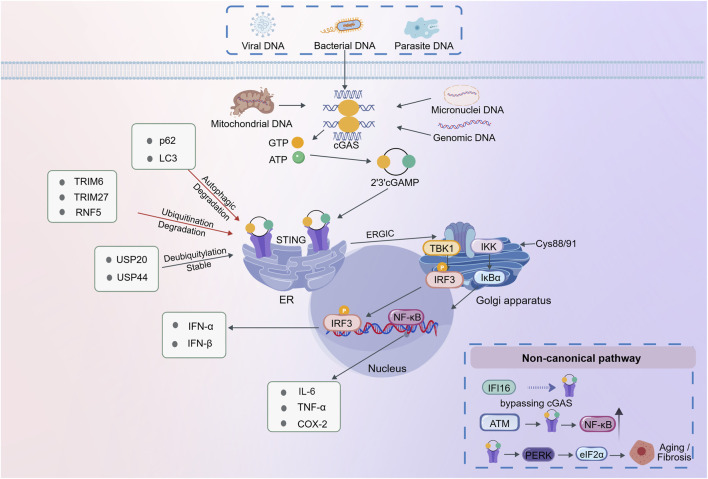
Molecular mechanism and signal transduction of the cGAS–STING pathway. This figure illustrates the core steps of cGAS–STING pathway activation. Cytosolic double-stranded DNA derived from multiple sources—including viral DNA, bacterial DNA, and parasite DNA (exogenous) and mitochondrial DNA, micronuclei DNA, and genomic DNA (endogenous self-DNA)—is recognized by the DNA sensor cGAS. Upon binding to DNA, cGAS catalyzes the synthesis of the second messenger 2′3′-cGAMP from ATP and GTP. 2′3′-cGAMP binds to the adaptor protein STING on the ER, triggering its sequential translocation through the ERGIC to the Golgi apparatus. At the Golgi, STING recruits and activates TBK1, which phosphorylates IRF3; STING also engages the IKK complex via Cys88/91, leading to IκBα degradation and NF-κB nuclear translocation. Within the nucleus, IRF3 and NF-κB drive expression of type I interferons (IFN-α/β) and pro-inflammatory cytokines (IL-6, TNF-α, and COX-2). Pathway activity is regulated by USP20/USP44-mediated deubiquitination (stabilization) and TRIM6/TRIM27/RNF5-mediated ubiquitination degradation; p62/LC3-mediated autophagic degradation provides an additional regulatory layer. The lower right panel illustrates three non-canonical activation pathways: IFI16-mediated STING activation bypassing cGAS (dashed arrow); ATM-mediated direct STING activation leading to preferential NF-κB signaling; and the STING–PERK–eIF2α axis linking ER stress to translational reprogramming, senescence, and fibrosis.

Under homeostatic conditions, cGAS is localized in both the nucleus and the cytoplasm; however, its enzymatic activity is tightly regulated to prevent aberrant immune responses against self-DNA. Within the nucleus, cGAS is maintained in an inactive state through chromatin binding. Specifically, histones (particularly the H2A–H2B complex) interact with the DNA-binding domain of cGAS, competitively inhibiting its erroneous activation by DNA (Ka et al., 2023). Conversely, upon the appearance of aberrant DNA in the cytoplasm—for instance, released from mitochondrial damage or genomic instability—the DNA-binding domain of cGAS rapidly recognizes and tightly binds to double-stranded DNA (dsDNA). This binding induces a conformational change in cGAS, exposing its catalytic center (Zhou et al., 2023). The activated cGAS subsequently catalyzes the conversion of ATP and GTP into a unique cyclic dinucleotide, 2′3′-cGAMP, which was the first cyclic dinucleotide identified in mammalian cells to function as a second messenger (Shen et al., 2025). Although cGAS activation is a rapid and effective process, its enzymatic activity is governed by a more intricate network of post-translational modifications (Figure 1).

To preserve immune homeostasis and prevent hyperactivation of the immune response against self-DNA, the enzymatic activity of cGAS is precisely controlled by a diverse array of post-translational modifications. These modifications collectively establish a complex regulatory network. For instance, SUMOylation primarily promotes the nuclear localization of cGAS, whereas phosphorylation, ubiquitination, and acetylation mainly modulate its protein stability and enzymatic activity (Shim et al., 2025). Specifically, AKT kinase-mediated phosphorylation of cGAS at Ser291 suppresses its enzymatic activity, while ULK1 kinase-mediated phosphorylation at Ser366 facilitates its degradation via the autophagic pathway (Jiang H. et al., 2025). The intricate interplay of these positive and negative regulatory mechanisms effectively maintains the low basal activity of cGAS, thereby preventing aberrant immune responses to self-DNA and preserving immune homeostasis (Shim et al., 2025). Ultimately, this diverse array of post-translational modifications ensures that the immune response is both timely and appropriately calibrated.

### 2.2 Structure and activation of STING

STING is an approximately 42 kDa endoplasmic reticulum (ER) membrane protein encoded by the *TMEM173* gene and plays a central role in the innate immune response. Its molecular structure is highly conserved and modular, primarily comprising four key domains: an N-terminal four-transmembrane domain responsible for anchoring STING to the ER membrane; a central dimerization domain that facilitates the stable existence of STING as a homodimer; a C-terminal ligand-binding domain (LBD) oriented toward the cytosol to sense and bind activating signals; and a C-terminal tail (CTT) that serves as an effector platform for downstream signal transduction (Hoong et al., 2020; Cai et al., 2024). In its resting state, the STING dimer localizes to the ER membrane via its transmembrane domains, with its LBD exposed to the cytosol, poised to receive upstream activation signals.

Classical STING activation is initiated upon binding of its endogenous ligand, 2′3′-cyclic GMP-AMP (cGAMP). This second messenger is synthesized by the DNA sensor cGAS following recognition of cytosolic double-stranded DNA. As a high-affinity ligand, the binding of 2′3′-cGAMP to the STING LBD induces a significant conformational change. This allosteric effect not only promotes STING translocation from the ER to the Golgi apparatus but also, importantly, makes this trafficking process a requisite step for achieving full STING activation (Shen et al., 2025). At the Golgi, STING recruits and activates TANK-binding kinase 1 (TBK1) via its C-terminal tail. Activated TBK1 subsequently phosphorylates STING at Ser366, an event that is critical for the formation of a stable STING-TBK1 signalosome platform and for ensuring the precise propagation of downstream signaling.

STING activation and the regulation of signal strength are further finely tuned by various post-translational modifications. Among these, palmitoylation is a crucial step, predominantly occurring at residues Cys88 and Cys91. This modification promotes STING aggregation and signal complex formation at the Golgi, enhancing signal transduction. Additionally, ubiquitination plays a significant role. For instance, K63-linked ubiquitination mediated by the E3 ubiquitin ligases TRIM56 and TRIM32 facilitates STING activation and signalosome assembly. Conversely, K48-linked ubiquitination, mediated by ligases such as RNF5, targets STING for proteasomal degradation, thereby terminating signaling in a timely manner to prevent overactivation (Yue et al., 2025). This precise spatiotemporal regulation ensures that STING signals are both efficiently initiated and promptly terminated, maintaining immune homeostasis.

Fully activated STING, via its C-terminal tail, acts as a signaling hub that orchestrates the activation of multiple downstream pathways. On one hand, STING functions as a scaffold protein, promoting the recruitment and phosphorylation of the transcription factor interferon regulatory factor 3 (IRF3) by TBK1. Phosphorylated IRF3 then forms dimers and translocates to the nucleus, initiating the expression of type I interferons (IFN-α/β). On the other hand, STING can independently activate the IκB kinase (IKK) complex, subsequently triggering the NF-κB pathway and inducing the production of a range of pro-inflammatory cytokines. It is through this dual signaling capability—simultaneously activating both the IRF3 and NF-κB axes—that STING effectively coordinates multiple facets of the innate immune response, establishing a robust anti-tumor or anti-infection state.

### 2.3 Downstream signaling pathways

The ultimate activation of the cGAS–STING pathway initiates and orchestrates the innate immune response through its downstream signaling network, a process primarily governed by two parallel signaling axes: the TBK1–IRF3 axis and the NF-κB axis (Figure 1).

The TBK1–IRF3 signaling axis constitutes the core pathway mediating type I interferon production. Activated STING recruits and activates TBK1, which, in turn, phosphorylates multiple serine residues (e.g., Ser386 and Ser396) at the C-terminus of the transcription factor IRF3. Phosphorylation at these sites induces conformational changes in IRF3, exposing its DNA-binding domain and nuclear localization signal, thereby promoting IRF3 dimerization and nuclear translocation (Schmid et al., 2024; Yum et al., 2021). Within the nucleus, IRF3 dimers bind to coactivators and specifically interact with the promoter regions of type I interferon genes, such as IFN-β, potently initiating their transcription (Schmid et al., 2024). Thus, the TBK1–IRF3 axis represents a critical branch of STING-induced antiviral immunity, although the downstream signaling output of STING extends far beyond this pathway.

In addition to IRF3, STING activation also robustly engages the NF-κB pathway. The C-terminal tail of STING contains a conserved motif that interacts with the IKK complex (comprising IKKα and IKKβ). Activated STING facilitates the recruitment of the IKK complex, promoting the phosphorylation and subsequent proteasomal degradation of IκBα. The degradation of IκBα releases the sequestered NF-κB (typically the p50/p65 heterodimer), enabling its translocation into the nucleus to initiate the transcription of a suite of pro-inflammatory cytokines and effector molecules, including interleukin-6 (IL-6), tumor necrosis factor-alpha (TNF-α), and cyclooxygenase-2 (COX-2) (Yum et al., 2021; Du et al., 2021). These two signaling pathways do not operate in isolation; rather, they exhibit significant crosstalk and synergistic interaction. Together, they orchestrate the magnitude, duration, and character of the innate immune response, ensuring an effective yet measured reaction to danger signals. Consequently, the coordinated activation of these two axes dictates the breadth and depth of the immune response downstream of STING.

The transcriptional program induced by STING activation forms a complex network whose effector molecules primarily include type I interferons (e.g., IFN-α and IFN-β), interferon-stimulated genes (ISGs), and a variety of chemokines and pro-inflammatory cytokines (Tian et al., 2022). These effector molecules act in concert to coordinate local and systemic immune responses. First, IFN-β, as a central effector, acts in an autocrine and paracrine manner on the producing cell and its neighbors. By binding to the cell surface type I interferon receptor (IFNAR), type I interferons activate the canonical JAK–STAT signaling pathway, subsequently inducing the expression of hundreds of ISGs. The proteins encoded by these ISGs possess broad antiviral, anti-proliferative, and immunomodulatory functions, establishing an “antipathogen state” within cells. Second, pathway activation induces the production of chemokines, such as CCL5 and CXCL10, which play a critical role in recruiting immune cells to sites of inflammation, including the tumor microenvironment. This recruitment helps “attract” immune cells, facilitating the initiation of adaptive immune responses (Tian et al., 2022; Yan et al., 2022). In summary, the cGAS–STING pathway, through its two core axes—TBK1–IRF3 and NF-κB—translates the recognition of cytosolic DNA into a complex transcriptional reprogramming event. This effectively orchestrates a multi-layered, multi-cellular cascade that bridges innate and adaptive immunity, laying the theoretical foundation for subsequent exploration of its significance in disease pathogenesis and therapeutic intervention.

### 2.4 Negative regulatory mechanisms of the pathway

The activity of the cGAS–STING pathway must be tightly regulated to prevent excessive inflammation and damage to self-tissues. Cells have evolved multi-layered negative regulatory mechanisms to ensure that immune responses are timely and appropriate. Dysregulation of this pathway is not only associated with autoimmune diseases, but tumor cells also frequently exploit these regulatory mechanisms to achieve immune evasion (Shim et al., 2025; Sasaki et al., 2023). The complexity of these regulatory mechanisms suggests that a profound understanding of the specific role of each node can help reveal the pathogenesis of related diseases and identify potential intervention targets.

First, at the cGAS level, various proteasomal pathways mediate its degradation to regulate protein stability. For instance, the autophagy receptor p62 recognizes and targets cGAS for autophagic degradation, whereas the E3 ubiquitin ligase TRIM6 promotes its proteasomal degradation by catalyzing K27-linked polyubiquitination of cGAS, thereby inhibiting pathway activity (Lu et al., 2023; Niu et al., 2025). Conversely, other molecules, such as the E3 ubiquitin ligase TRIM14, stabilize the cGAS protein by recruiting the deubiquitinase USP14 to remove K48-linked ubiquitin chains from cGAS, preventing its excessive degradation and maintaining basal levels (Lu et al., 2023). Furthermore, post-translational modifications directly regulate cGAS enzymatic activity; for example, AKT kinase-mediated phosphorylation of cGAS at Ser291 inhibits its ability to synthesize cGAMP. This regulatory mechanism is particularly pronounced in tumor cells with active growth factor signaling and may explain the suppressed cGAS activity observed in some tumors (Zhang et al., 2024a). Thus, cGAS, as the initiating sensor of the pathway, has its protein stability and enzymatic activity finely tuned by multiple factors. Next, we will observe that the regulation of the downstream adaptor protein STING is even more complex, involving multiple layers of regulation, including subcellular localization and degradation.

Second, at the level of the key adaptor protein STING, its trafficking from the ER to the Golgi apparatus and subsequent degradation are central regulatory nodes. ER-resident proteins such as STIM1 and CALR can restrict STING trafficking and activation. Following STING activation, various E3 ubiquitin ligases, including RNF5, TRIM30α, and AMFR, mediate its ubiquitination and degradation, thereby terminating signal transduction and preventing signal amplification. Of particular note, the autophagy pathway plays a critical role in STING degradation, forming a complex interplay between cGAS–STING signaling and autophagy. On one hand, activated STING can be directly recruited to autolysosomes for degradation via interaction with LC3 (Gan et al., 2021). On the other hand, autophagy also reduces pathway activation at its source by clearing damage-associated molecular patterns such as cytosolic DNA and dysfunctional mitochondria (Schmid et al., 2024; Lu et al., 2023). This mechanism not only terminates STING signaling but also eliminates activated STING protein, which is crucial for maintaining immune homeostasis and preventing persistent inflammation. However, beyond these mechanisms acting directly on signaling molecules, cells also possess key regulators that inhibit pathway activation at the source, the most representative of which is the nuclease TREX1.

In addition, the nuclease TREX1 plays a unique and critical “braking” role in regulating the cGAS–STING pathway. TREX1, localized to the ER, is the primary exonuclease responsible for degrading cytosolic DNA. By efficiently clearing aberrant DNA fragments that enter the cytoplasm, TREX1 greatly reduces the availability of ligands recognizable by cGAS, thus exerting a key inhibitory function upstream of the pathway. Loss-of-function mutations in TREX1 lead to massive accumulation of cytosolic DNA and hyperactivation of the cGAS–STING pathway, which is the main pathogenic mechanism in severe autoinflammatory diseases such as Aicardi–Goutières syndrome (Wang Q. et al., 2022). In tumor biology, upregulation of TREX1 expression can effectively suppress cGAS–STING pathway activation, thereby helping tumor cells evade immune surveillance and representing a significant mechanism of acquired immune resistance in cancer (Wang Q. et al., 2022). For instance, while radiotherapy induces DNA damage, it can also upregulate TREX1 expression in tumor cells, thereby limiting the anti-tumor immune effects potentially triggered by the radiation itself. As a crucial “sentinel” upstream of the pathway, TREX1’s importance is clear. Beyond these major regulatory nodes, a more complex regulatory network composed of metabolic and deubiquitination factors further shapes pathway activity.

Beyond the primary regulatory nodes mentioned above, various other mechanisms collectively shape the intensity and duration of pathway activation. For example, metabolites and lipid metabolism are also involved: increased cholesterol biosynthesis induced by radiotherapy has been found to retain STING protein in the ER, inhibiting its activation and downstream interferon signaling and contributing to therapeutic resistance in tumors (Zhu L. et al., 2025). Furthermore, certain deubiquitinating enzymes, such as USP20 and USP44, remove K48-linked ubiquitin chains from STING, thereby stabilizing the protein, while protein phosphatases, such as PP1 and PP2A, negatively regulate signaling by dephosphorylating key kinases or the transcription factor IRF3. These multi-layered and multi-node regulatory networks intertwine to ensure the precise spatiotemporal activation of the cGAS–STING pathway and maintain immune homeostasis. Tumor cells often hijack these regulatory mechanisms to achieve immune evasion. A comprehensive and in-depth dissection of this complex regulatory network will provide a crucial theoretical foundation for the development of novel immunotherapies targeting autoimmune diseases and cancer.

## 3 Dual role of the cGAS–STING pathway in cancer

### 3.1 Tumor-suppressive functions

**FIGURE 2:**
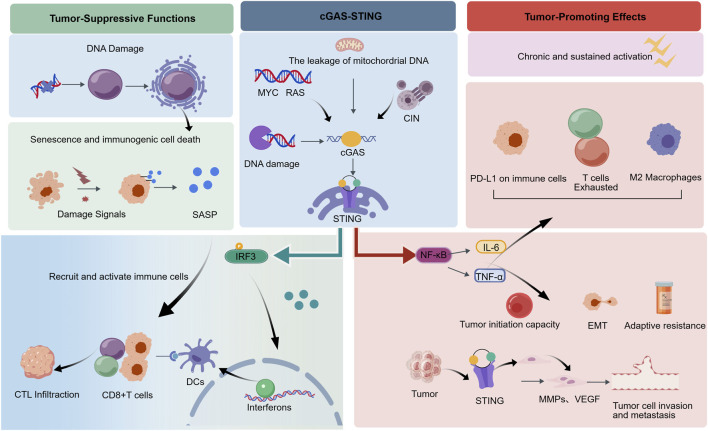
Dual role of the cGAS–STING pathway in cancer. This figure depicts the context-dependent dual functions of the cGAS–STING pathway in oncology. (Left, tumor-suppressive role) Acute activation, typically triggered by therapy-induced DNA damage, leads to a robust type I interferon response via IRF3. This promotes dendritic cell maturation and cross-presentation, subsequently eliciting the priming and infiltration of CD8^+^ cytotoxic T lymphocytes, resulting in effective tumor cell killing and immunological memory. (Right, tumor-promoting role) In contrast, chronic activation driven by persistent chromosomal instability predominantly signals through the NF-κB pathway, inducing IL-6 and TNF-α production. This promotes tumor initiation capacity, EMT, and adaptive therapy resistance. Chronic activation further drives PD-L1 upregulation on immune cells, leading to T-cell exhaustion, and promotes the recruitment of M2 macrophages and other immunosuppressive populations, shaping an immunosuppressive TME that facilitates tumor cell invasion and metastasis.

The cGAS–STING pathway, as a critical component of the innate immune system, plays an indispensable role in tumor immunosurveillance (Figure 2). By sensing tumor-derived DNA, this pathway initiates downstream signaling cascades, thereby exerting inhibitory effects at various stages of tumorigenesis (Samson and Ablasser, 2022). A substantial body of research has confirmed that this pathway synergistically suppresses tumor initiation and progression through multiple mechanisms. Herein, we systematically elucidate the core functions of the cGAS–STING pathway in anti-tumor immunity, examining upstream activating events, downstream effector molecules, regulation of cellular fate, and the involvement of immune cells.

First, DNA damage serves as the primary upstream event that triggers the activation of the cGAS–STING pathway. In the early stages of tumorigenesis, the activation of oncogenes (such as MYC or RAS) can induce replication stress, which subsequently leads to DNA damage and the formation of cellular structures such as micronuclei or chromatin bridges. The rupture of the nuclear envelope associated with these structures allows genomic DNA to leak into the cytoplasm, where it is recognized by the DNA sensor cGAS, thereby initiating immunosurveillance (Zheng et al., 2020; Shen et al., 2021). Furthermore, the leakage of mitochondrial DNA (mtDNA) represents another significant source of pathway activation, particularly in the context of mitochondrial dysfunction (Xia et al., 2025). Consequently, this DNA-sensing mechanism, driven by genomic instability, is considered a crucial line of defense against tumors at their nascent stage (Beernaert and Parkes, 2023). However, the activation of the cGAS–STING pathway is merely the initial step in immunosurveillance; how it subsequently mobilizes the immune system to exert anti-tumor effects depends on the amplification of downstream signals.

Second, the activation of the cGAS–STING pathway exerts its core anti-tumor effects primarily through the production of type I interferons (IFN-α/β) (Kwon and Bakhoum, 2020). Type I interferons not only directly inhibit tumor cell proliferation and promote their apoptosis but also, more importantly, remodel the tumor microenvironment, particularly by acting on DCs. Type I interferons act on DCs to enhance their maturation and antigen-processing machinery, enabling efficient cross-presentation of tumor-derived antigens and the subsequent priming of tumor-specific CD8^+^ T cells (Jiang et al., 2020; Moussion and Delamarre, 2024). This process establishes a critical link between innate and adaptive immunity, which is essential for generating a durable anti-tumor effect. Beyond the adaptive immunity mediated by type I interferons, the cGAS–STING pathway can also act directly on tumor cells, inducing senescence or immunogenic cell death and thereby further potentiating the anti-tumor response.

Furthermore, this pathway can induce senescence and immunogenic cell death in tumor cells. Research indicates that sustained cGAS–STING signaling can drive tumor cells into a senescent state, accompanied by the development of a senescence-associated secretory phenotype (SASP) (Loo et al., 2020). The SASP comprises a diverse array of pro-inflammatory cytokines and chemokines that can broadly recruit and activate immune cells. Notably, while some factors within the SASP (e.g., IL-6 and IL-8) may promote inflammation in certain contexts, the immunomodulatory SASP triggered by cGAS–STING generally contributes to enhancing anti-tumor immune responses and facilitates the immune-mediated clearance of senescent tumor cells (Yue et al., 2025). Moreover, activation of this pathway is also closely linked to other forms of immunogenic cell death, further amplifying the anti-tumor immune effect (Du et al., 2021). It is important to note that the anti-tumor functions of the cGAS–STING pathway are not limited to its effects within tumor cells; its activation in tumor-infiltrating immune cells also plays a pivotal role in shaping an anti-tumor immune microenvironment.

In addition, cGAS–STING activation in tumor-infiltrating immune cells is critically important. Dendritic cells and macrophages, for instance, can activate their own STING pathway by taking up tumor-derived DNA or acquiring cGAMP released from tumor cells via gap junctions. This bystander activation effect prompts these immune cells to produce IFN-β and other pro-inflammatory cytokines, further shaping and sustaining a robust anti-tumor immune microenvironment (Kabashima et al., 2022; Pépin et al., 2017). In mouse tumor models, the loss of STING directly results in reduced tumor infiltration by CD8^+^ T cells and accelerated tumor growth, providing compelling evidence for the central role of this pathway in mediating anti-tumor immunity (Moussion and Delamarre, 2024). The findings from these fundamental studies are corroborated by clinical samples, further underscoring the critical status of the cGAS–STING pathway in tumor immunity.

Finally, a substantial body of clinical research provides strong support for the tumor-suppressive functions of the cGAS–STING pathway. In various tumor types, high expression of cGAS or STING positively correlates with favorable patient prognosis (Chen et al., 2024; An et al., 2019). Specifically in colorectal cancer, microsatellite instability-high (MSI-H) tumors frequently exhibit high expression of cGAS and STING, along with abundant CD8^+^ T-cell infiltration (Kunac et al., 2022; Kaneta et al., 2022). This may represent one of the intrinsic reasons for the favorable response of such tumors to immune checkpoint inhibitor therapy, further highlighting the pivotal role of the cGAS–STING pathway in tumor immunity (Eksin, 2022). Clarifying how these regulatory mechanisms operate, together with exploring rational combination strategies that target them, will help open new avenues for cancer immunotherapy.

### 3.2 Tumor-promoting effects

Although the cGAS–STING pathway plays a critical role in initiating anti-tumor immunity, a growing body of research in recent years has revealed that it can be “hijacked” under specific conditions, particularly during the advanced stages of tumor progression, to instead promote tumor initiation, development, and metastasis, revealing a striking context-dependent shift in the pathway’s overall function (Samson and Ablasser, 2022; Kwon and Bakhoum, 2020; Shen et al., 2025). This tumor-promoting effect is not attributable to a single mechanism; rather, it is synergistically achieved through the establishment of chronic inflammation, the shaping of an immunosuppressive microenvironment, and the activation of pro-survival signaling networks intrinsic to tumor cells (Figure 2).

#### 3.2.1 Chronic low-grade inflammation: the switch from immune surveillance to inflammation-driven cancer promotion

Chronic low-grade inflammation serves as a key driver of the functional shift in the cGAS–STING pathway from anti-tumor to pro-tumor activities. During tumor progression, persistent chromosomal instability (CIN) leads to the accumulation of numerous micronuclei and cytoplasmic chromatin fragments, which, in turn, trigger chronic and sustained activation of the cGAS–STING pathway (Beernaert and Parkes, 2023; Gulen et al., 2023). In contrast to the robust protective immunity elicited by acute, transient activation, chronic STING signaling predominantly relies on the activation of the NF-κB pathway, leading to the sustained release of a cascade of pro-inflammatory cytokines, including IL-6 and TNF-α (Wheeler and Unterholzner, 2023). To understand this functional transition, it is crucial to distinguish the profoundly different biological outcomes resulting from acute versus chronic activation of the cGAS–STING pathway. This inflammatory milieu, driven by chronic STING signaling, provides a fertile soil for tumor evolution, fostering development through multiple mechanisms. First, inflammatory cytokines can induce the generation of reactive oxygen species (ROS), leading to persistent DNA damage and gene mutations, thereby accelerating the genetic evolution of the tumor. Second, these factors can act directly on tumor cells, activating survival pathways such as STAT3 signaling to enhance their proliferation and resistance to apoptosis. Furthermore, chronic inflammation can promote the expression of vascular endothelial growth factor (VEGF), driving angiogenesis and creating conditions conducive to epithelial–mesenchymal transition (EMT), which, in turn, enhances local tumor invasion and distant metastatic capacity (Loo et al., 2020; Wang S. L. et al., 2023). Within this microenvironment, inflammatory factors act synergistically through multiple pathways, accelerating tumor cell evolution and survival while remodeling the tumor stroma to promote angiogenesis and the acquisition of an invasive, metastatic phenotype. Consequently, the chronic inflammation mediated by the cGAS–STING pathway, through its concerted actions on both the tumor cells themselves and their microenvironment, forms a complex network that facilitates the multifaceted malignant progression of cancer.

#### 3.2.2 Shaping an immunosuppressive microenvironment: promoting tumor immune evasion

Chronic activation of the cGAS–STING pathway not only directly fuels inflammatory responses but also profoundly remodels the tumor microenvironment, driving its conversion toward an immunosuppressive phenotype, which ultimately facilitates tumor immune evasion. Research has demonstrated that type I interferons (e.g., IFN-β) induced by persistent STING signaling can, to some extent, upregulate the expression of co-inhibitory molecules such as PD-L1 on immune cells, including dendritic cells, macrophages, and even tumor cells themselves (Gan et al., 2021; Liang et al., 2024). This adaptive upregulation represents a key mechanism by which tumors resist T-cell-mediated attacks. In addition, sustained STING signaling promotes the polarization of tumor-associated macrophages (TAMs) toward an immunosuppressive M2 phenotype and facilitates the recruitment and expansion of regulatory T cells (Tregs) and myeloid-derived suppressor cells, collectively contributing to the formation of a functional immunological barrier (Jacoberger-Foissac et al., 2023). In tumors characterized by high chromosomal instability, persistent cGAS–STING signaling has been paradoxically associated with reduced infiltration and functional exhaustion of CD8^+^ T cells, suggesting that during immunoediting, tumors may exploit chronic pathway activity to select and consolidate a microenvironment conducive to immune evasion (Sokač et al., 2022). Therefore, a thorough understanding of the dual role of the cGAS–STING pathway in tumor progression is critical for the development of precision immunotherapeutic strategies targeting this pathway.

#### 3.2.3 Conferring intrinsic malignant phenotypes: enhanced stemness, survival, and therapy resistance

Beyond its role in sculpting the tumor microenvironment, the activation of cGAS–STING signaling intrinsically within tumor cells can directly contribute to more aggressive malignant behaviors. First, studies have confirmed that STING activation can sustain or enhance cancer stem cell properties by activating transcription factors such as STAT3 and NF-κB, thereby increasing tumor initiation capacity and metastatic potential (Liu F. R. et al., 2022). Second, activation of this pathway can upregulate the expression of EMT-related transcription factors, including Snail and Twist, thereby promoting the acquisition of migratory and invasive capabilities. More importantly, while DNA-damaging therapies such as chemotherapy and radiotherapy effectively kill tumor cells, they also inadvertently activate the cGAS–STING pathway within the surviving cancer cells. This cancer cell-autonomous STING activation can, paradoxically, endow these cells with the capacity to resist therapeutic stress by upregulating pro-survival genes and enhancing DNA repair mechanisms, thereby contributing to so-called “adaptive resistance” (Zhu L. et al., 2025; Lv et al., 2023). This suggests that when employing STING agonists in combination with chemotherapy, careful consideration must be given to the potential risk of promoting therapy resistance.

#### 3.2.4 Intercellular transmission of cGAMP: amplifying pro-tumorigenic signaling

The intricate crosstalk within the tumor microenvironment significantly amplifies the overall impact of the cGAS–STING pathway. The second messenger cGAMP, generated by tumor cells, can be transferred to adjacent stromal cells—including cancer-associated fibroblasts (CAFs), endothelial cells, and immune cells—via gap junctions or within encapsulated vesicles (Pépin et al., 2017; Wu et al., 2023). This intercellular communication is particularly consequential in CAFs: upon receiving cGAMP, STING activation drives their transdifferentiation toward a pro-inflammatory phenotype. These activated CAFs subsequently secrete a spectrum of cytokines and matrix-remodeling enzymes, such as matrix metalloproteinases (MMPs), thereby facilitating tumor cell invasion and metastasis. Notably, a study in ovarian cancer revealed that chemotherapeutic agents such as cisplatin can induce tumor cells to transfer DNA to CAFs. This activates the cGAS–STING–IFNB1 pathway in fibroblasts, leading to IFN-β secretion, which ultimately enhances the resistance of tumor cells to platinum-based drugs (Liu et al., 2024). This finding provides clear evidence for the critical role of tumor–stroma crosstalk in the acquisition of therapy resistance.

#### 3.2.5 Regulation of tumor metastasis: a dynamic, stage-dependent process

The cGAS–STING pathway plays an exceptionally complex and even paradoxical role in tumor metastasis (Ng et al., 2018). On one hand, within the circulatory system, isolated tumor cells (circulating tumor cells, CTCs) are subjected to mechanical stresses, such as hemodynamic shear forces, which can cause nuclear envelope rupture and the release of genomic DNA, thereby activating the cGAS–STING pathway. The resulting IFN-β can activate immune cells, such as natural killer (NK) cells, to eliminate these potential metastatic “seeds,” thereby exerting a metastasis-suppressive function (Frittoli et al., 2023). On the other hand, within established metastatic lesions, sustained cGAS–STING signaling can promote immune evasion and metastatic outgrowth through the aforementioned mechanisms, including the shaping of an immunosuppressive microenvironment and the maintenance of tumor cell stemness. Furthermore, STING signaling can facilitate tumor cell dissemination by promoting angiogenesis and lymphangiogenesis, thereby creating “pathways” for metastatic spread, and can also directly induce the EMT, enhancing the ability of tumor cells to detach from the primary tumor. Consequently, the ultimate impact of the cGAS–STING pathway on metastasis is dynamically regulated, contingent upon the specific stage of the tumor cell and the precise state of its microenvironment.

Beyond the canonical mechanisms described above, emerging evidence has unveiled critical non-canonical activation pathways and pro-tumorigenic signaling axes that significantly expand the functional scope of the cGAS–STING pathway in tumor biology. In response to replication stress and DNA damage, the ATM kinase can directly activate STING independent of cGAS, often involving the DNA sensor IFI16, leading to a predominant NF-κB-driven inflammatory response rather than a strong type I interferon response (Kwon and Bakhoum, 2020). This ATM/IFI16-mediated non-canonical pathway represents a mechanistically distinct route of STING activation with context-dependent pro- or anti-tumorigenic consequences. Furthermore, a cGAS–STING–PERK–eIF2α axis has been identified that operates independently of IRF3 and TBK1, linking ER stress and translational control to STING activation, thereby influencing senescence, autophagy, and fibrosis within the tumor microenvironment (Vasiyani, 2026). Beyond these activation axes, a key pro-tumorigenic mechanism involves STING-dependent induction of IL-6, which activates the STAT3 signaling pathway. This IL-6–STAT3 axis promotes cancer cell survival, stemness, and resistance to therapy, particularly in aggressive subtypes such as triple-negative breast cancer (TNBC) (Liu L. C. et al., 2022). Pharmacological targeting of this downstream effector axis, for instance, using JAK/STAT inhibitors to block IL-6–STAT3 signaling, represents a rational combination strategy to overcome therapy resistance driven by chronic STING activation (Table 1).

**TABLE 1:** Key mechanisms of non-canonical and pro-tumorigenic cGAS–STING signaling.

Pathway/Mechanism	Key component	Functional outcome in tumor biology	Key reference
ATM/IFI16 non-canonical activation	ATM kinase; DNA sensor IFI16; STING (cGAS-independent)	Predominantly activates NF-κB rather than IRF3; drives pro-inflammatory cytokine production independent of type I IFN response; context-dependent pro- or anti-tumorigenic outcomes	Kwon and Bakhoum (2020)
cGAS–STING–PERK–eIF2α axis	STING; PERK kinase; eIF2α; independent of TBK1/IRF3	Links ER stress to STING activation; controls translational programs; promotes senescence, autophagy, and organ fibrosis in the tumor microenvironment	Vasiyani (2026)
STING–IL-6–STAT3 pro-tumorigenic axis	STING; IL-6; STAT3; JAK kinases	Drives cancer cell survival, stemness, and chemoresistance; particularly active in TNBC; pharmacological blockade with JAK/STAT inhibitors can overcome therapy resistance	Liu L. C. et al. (2022)
Chronic NF-κB inflammatory signaling	Persistent low-level STING activation; NF-κB; IL-6, TNF-α, and IL-8	Fuels tumor-promoting chronic inflammation; recruits MDSCs and Tregs; promotes EMT, angiogenesis, and metastasis; shapes immunosuppressive TME	Samson and Ablasser (2022)

### 3.3 Determinants of functional duality

The cGAS–STING pathway exhibits significant functional duality in cancer, where its ultimate outcome—whether pro- or anti-tumorigenic—hinges on a delicate balance among multiple factors. These determinants primarily encompass signal dynamics, activation intensity, cell type specificity, tumor genetic background, and characteristics of the tumor microenvironment.

First, signal dynamics represent a pivotal determinant of the functional direction of the cGAS–STING pathway, with the core concept centered on “spatiotemporal regulation.” Specifically, acute and transient STING activation predominantly induces a type I interferon response, thereby promoting DC maturation and T-cell activation, which culminates in potent anti-tumor immunity (Samson and Ablasser, 2022). In stark contrast, chronic, low-intensity STING signaling—often stemming from persistent DNA damage or sustained stimulation by low levels of self-DNA—tends to continuously fuel the production of inflammatory cytokines such as TNF-α and IL-6 via the NF-κB pathway. This state of chronic inflammation not only fails to effectively engage adaptive immunity but actively sculpts an immunosuppressive TME by recruiting MDSCs and Tregs, thereby paradoxically facilitating tumor progression (Samson and Ablasser, 2022; Kwon and Bakhoum, 2020). This insight has important implications for the design of therapies based on STING agonists, underscoring the need to precisely control pathway activation kinetics to achieve optimal therapeutic outcomes.

Second, the intensity of activation profoundly influences the functional output of STING signaling. Research indicates that varying stimulus strengths can dictate vastly different cellular fates and immunological consequences (Wang et al., 2020). For instance, strong STING signals can induce apoptosis or pyroptosis in tumor cells; these forms of immunogenic cell death (ICD) release tumor antigens and danger signals, thereby potentiating anti-tumor immunity (Yue et al., 2025). Conversely, moderate STING signaling primarily drives cytokine production and immunoregulatory functions, thereby promoting antigen presentation and T-cell priming. However, excessively weak signals may fail to adequately ignite an immune response and could, under certain circumstances, induce immune tolerance, enabling tumor cells to evade immunosurveillance. This paradigm explains why STING agonists of different strengths or types can yield divergent therapeutic outcomes in preclinical studies.

Furthermore, cell-type specificity constitutes another crucial determinant. The functional heterogeneity of the cGAS–STING pathway across different cell types significantly shapes the overall anti-tumor effect (Wang L. et al., 2026). For example, in tumor cells, STING activation can directly trigger apoptosis or senescence, while simultaneously producing chemokines (such as CCL5 and CXCL10) to recruit immune cells. In dendritic cells, STING signaling promotes their maturation, antigen uptake, and cross-presentation capabilities, serving as a critical bridge between innate and adaptive immunity (Lanng et al., 2024). In macrophages, STING signals can induce polarization toward the anti-tumor M1 phenotype, yet under specific conditions, they may also facilitate conversion to the immunosuppressive M2 phenotype (Jacoberger-Foissac et al., 2023). Collectively, these cell-type-specific responses determine the net effect of the cGAS–STING pathway within the complex ecosystem of the tumor microenvironment.

It is noteworthy that the tumor type and genetic background significantly influence the functional status and biological outcomes of the cGAS–STING pathway. In colorectal cancer, for instance, the pathway is typically highly active in microsatellite instability-high (MSI-H) tumors due to the accumulation of substantial cytosolic DNA resulting from frequent DNA replication errors. This high activity is closely associated with abundant immune cell infiltration within the tumor tissue and favorable responses to immune checkpoint inhibitors (Kunac et al., 2022; Zhang et al., 2024b). In contrast, in the majority of microsatellite-stable (MSS) colorectal cancers, the pathway is frequently silenced by aberrant epigenetic modifications, such as promoter hypermethylation. This silencing contributes to the formation of an immune “cold” tumor phenotype and confers resistance to immunotherapy (Sasaki et al., 2023). Furthermore, the mutational status of p53 also profoundly modulates the functional output of this pathway. Mutant p53 proteins can alter the immune microenvironment landscape by, for example, suppressing STING signaling or enhancing NF-κB activation. This alteration can redirect STING signaling from immune activation toward a pro-inflammatory, tumor-promoting direction (Zhang et al., 2024c). Dissecting how tumor genetic background shapes cGAS–STING pathway output will therefore be essential for designing more precisely targeted immunotherapeutic strategies.

Finally, the composition and state of the tumor microenvironment represent crucial determinants that cannot be overlooked. Stress conditions within the tumor, such as hypoxia, acidosis, and nutrient deprivation, can significantly alter both the direction and intensity of cGAS–STING signaling (Zhang et al., 2024d). For example, the relative proportions of different immune cell subsets within the microenvironment—particularly the enrichment of regulatory T cells and myeloid-derived suppressor cells—can dictate whether STING activation ultimately leads to immune activation or immunosuppression. Additionally, stromal cells, especially cancer-associated fibroblasts, can suppress the expression and function of the cGAS–STING pathway in tumor cells or immune cells by secreting specific extracellular matrix (ECM) components and soluble factors such as TGF-β. This, in turn, influences the pathway’s overall role in tumor immunity (Heintzman et al., 2022). The interplay between these microenvironmental factors and the cGAS–STING pathway collectively shapes the tumor’s immune phenotype and its response to therapy. Consequently, a thorough investigation into the regulatory mechanisms of the cGAS–STING pathway within specific microenvironmental contexts will provide a crucial theoretical foundation for optimizing cancer immunotherapeutic strategies targeting this pathway.

## 4 Dysregulation of the cGAS–STING pathway in tumor cells

### 4.1 Genomic instability and sources of cytosolic DNA

**FIGURE 3:**
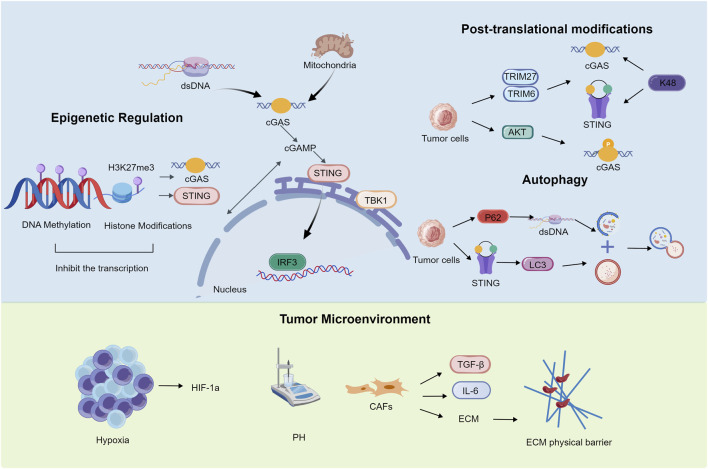
Mechanisms of cGAS–STING pathway dysregulation in tumor cells. Tumor cells employ multiple interconnected strategies to silence or evade the cGAS–STING pathway. (1) Epigenetic regulation: transcriptional silencing of cGAS and STING genes is achieved through promoter DNA hypermethylation and repressive histone modifications (e.g., H3K27me3). (2) Post-translational modifications: protein stability and activity are regulated by post-translational modifications. E3 ubiquitin ligases (e.g., TRIM6 and TRIM27) target cGAS and STING for K48-linked proteasomal degradation. Kinases such as AKT can directly phosphorylate cGAS, thereby inhibiting its enzymatic activity. (3) Autophagy: the autophagic machinery is hijacked to clear cytosolic DNA (via p62) and directly degrade activated STING protein (via LC3 interaction), thereby attenuating signal strength. (4) Tumor microenvironment: conditions such as hypoxia, acidosis, and paracrine signals from cancer-associated fibroblasts can suppress the expression or function of pathway components, further promoting immune evasion.

Genomic instability, a hallmark of cancer, serves as a critical upstream event that triggers the activation of the cGAS–STING pathway. CIN, for instance, leads to aberrant chromosome segregation during cell division, resulting in the formation of micronuclei. These micronuclei are prone to rupture due to their compromised nuclear envelope integrity, thereby exposing genomic DNA to the cytoplasm. This misplaced DNA is subsequently recognized by the cytosolic DNA sensor cGAS, initiating immune signal transduction (Samson and Ablasser, 2022; Kwon and Bakhoum, 2020). However, despite the frequent accumulation of cytosolic DNA in tumor cells exhibiting high CIN, the cGAS–STING pathway is often found to be silenced in these contexts. This apparent paradox suggests that tumors, during their evolutionary progression, have developed various escape mechanisms to circumvent immune surveillance, thereby enabling their survival and expansion even under conditions of profound genomic stress (Shim et al., 2025; Beernaert and Parkes, 2023). Consequently, while chromosomal instability provides a potential triggering signal for cGAS–STING, tumor cells can employ multiple strategies to block activation of this pathway, thereby sustaining their malignant growth advantage (Figure 3).

Beyond nuclear DNA, the release of mtDNA represents another significant trigger for cGAS–STING pathway activation. Mitochondria are not only central to cellular energy metabolism but also act as key organelles for sensing cellular stress. Under conditions such as oxidative stress, impaired mitophagy, or loss of mitochondrial membrane potential, mitochondrial structural integrity is compromised. This leads to the leakage of mtDNA into the cytoplasm, where it can provoke an innate immune response (Xia et al., 2025). Unlike nuclear DNA, mtDNA lacks protective histone packaging and possesses limited DNA repair capacity, rendering it more susceptible to damage and subsequent release under stressful conditions. Studies have confirmed that chemotherapeutic agents, reactive oxygen species, and mitochondrial toxins can all induce mtDNA leakage and consequently activate the cGAS–STING signaling pathway (Yan et al., 2022; Hu M. et al., 2021). Notably, the immunostimulatory capacity of mtDNA appears to be modulated by its oxidation state, with oxidized mtDNA exhibiting a more potent pro-inflammatory potential. This suggests a more complex role for mtDNA in immune regulation than previously appreciated (Xia et al., 2025; Li Z. et al., 2024). These findings broaden our understanding of mtDNA function in tumor immunity and highlight its potential as a novel target for therapeutic intervention.

Endogenous retroelements represent another potential source of cytosolic DNA. Endogenous retroviral elements are widely distributed throughout the human genome and are typically epigenetically silenced under normal physiological conditions. However, during aging, epigenetic dysregulation, or stress conditions, these elements can be reactivated, generating DNA through reverse transcription that accumulates in the cytoplasm (Lindholm et al., 2023). The re-expression of these retroelements, particularly in aged individuals, may be closely associated with elevated basal inflammation levels and an increased risk of tumorigenesis. Notably, one of the antitumor mechanisms of clinically used DNA-demethylating agents, such as decitabine and azacitidine, involves the activation of retroelements, leading to the accumulation of cytosolic DNA. This, in turn, triggers the cGAS–STING pathway and enhances tumor immunogenicity (Sasaki et al., 2023). Therefore, the regulation of retroelements not only impacts genomic stability but also presents a novel opportunity for cancer immunotherapy.

Defects in DNA damage repair also constitute a critical source of cytosolic DNA accumulation. For instance, deficiencies in homologous recombination repair caused by loss-of-function mutations in BRCA1/2, or impaired replication error repair due to mutations in DNA polymerase ε, can significantly exacerbate genomic damage and thereby promote the formation of cytosolic DNA (Huang and Zhou, 2021). Although such cells theoretically possess the prerequisites for cGAS–STING pathway activation, many tumors effectively block this pathway through various mechanisms to achieve immune evasion. For example, in BRCA1-deficient ovarian cancer, STING expression is frequently downregulated, consequently inhibiting the initiation of innate immune responses (Sasaki et al., 2023). These findings suggest that, under the immune pressure exerted by genomic instability, tumors both continuously generate cytosolic DNA and concurrently develop sophisticated immunoregulatory networks. This intricate balance between survival and immune evasion constitutes a core characteristic of tumor evolution and provides a more comprehensive perspective on understanding the tumor immune microenvironment.

### 4.2 Epigenetic regulation

Epigenetic regulation constitutes a core mechanism by which tumor cells suppress the cGAS–STING pathway. This encompasses a variety of complex and interconnected processes, including DNA methylation, post-translational histone modifications, and chromatin remodeling. These mechanisms allow tumor cells to reversibly silence the expression of key pathway genes without altering the underlying DNA sequence, thereby effectively evading host immune surveillance (Sasaki et al., 2023). The establishment of this multi-layered regulatory network endows tumor cells with the flexibility to adapt to dynamic immune pressures throughout their evolution.

DNA methylation is a prevalent mechanism responsible for the transcriptional silencing of the cGAS–STING pathway across numerous cancer types. Studies have demonstrated that the promoter regions of the STING and cGAS genes frequently exhibit aberrant hypermethylation in various tumors, including colorectal cancer and non-small cell lung cancer, directly leading to the suppression of their gene expression (Sasaki et al., 2023; Hao, 2023). For instance, in colorectal cancer, STING promoter methylation levels are significantly negatively correlated with both reduced STING protein expression and decreased infiltration of CD8^+^ T cells within tumor tissue. This epigenetic silencing not only diminishes tumor immunogenicity but also constitutes a major cause of primary or acquired resistance to immune checkpoint inhibitor therapies. Importantly, DNA methyltransferase inhibitors, such as decitabine, can effectively reverse this silenced state, restoring STING expression in tumor cells and significantly enhancing their sensitivity to STING agonists (Tu et al., 2026). This provides a robust theoretical foundation for combination therapeutic strategies that pair demethylating agents with STING agonists. Furthermore, the methylation status of the STING gene holds potential as a predictive biomarker for immunotherapy efficacy and for identifying patient populations most likely to benefit from such treatments. Therefore, targeting DNA methylation not only offers a strategy to remodel the tumor immune microenvironment but also presents a promising avenue for achieving personalized immuno-oncologic interventions.

In addition to DNA methylation, histone modifications also play a pivotal role in the fine-tuned regulation of the cGAS–STING pathway. On one hand, histone deacetylases (HDACs) inhibit the transcription of cGAS and STING by maintaining a compact chromatin state that restricts transcription factor binding. Correspondingly, treatment with HDAC inhibitors can remodel chromatin accessibility in these regions, upregulate the expression of these genes, and restore pathway activity (Wang et al., 2024a). On the other hand, specific histone methylations are also involved. For instance, the H3K27me3 modification mediated by the histone methyltransferase EZH2 is a canonical transcriptional repressive mark; its enrichment at the STING gene locus silences gene expression. Conversely, the use of EZH2 inhibitors can relieve this repression and demonstrate synergistic anti-tumor effects when combined with STING agonists. Furthermore, members of the KDM5 family of histone demethylases can inhibit the transcription of STING and downstream ISGs by removing H3K4me3 (a transcriptional activation mark), thereby promoting tumor immune evasion (He et al., 2021). These findings indicate that aberrant activity of histone-modifying enzymes is a significant driver of tumor immune evasion, and targeting these enzymes holds promise for restoring the immune-sensing function of the pathway through “epigenetic reprogramming.”

Chromatin remodeling complexes, particularly the SWI/SNF complex, play a decisive role in both basal and inducible expression of the cGAS–STING pathway by altering nucleosome positioning and chromatin accessibility. ARID1A, a key DNA-binding subunit of the SWI/SNF complex, frequently undergoes loss-of-function mutations in various cancers, including ovarian and gastric cancers (Zhu et al., 2024). Loss of ARID1A leads to global changes in chromatin accessibility. On one hand, this may impair high-fidelity DNA damage repair, resulting in genomic instability and aberrant accumulation of cytosolic DNA. On the other hand, this abnormal chromatin remodeling can paradoxically enhance the accessibility of transcription factors to cGAS–STING pathway-related gene loci, thereby upregulating their transcriptional activity under certain circumstances. Remarkably, clinical data show that patients with tumors harboring ARID1A mutations respond better to immune checkpoint inhibitor therapy, a phenomenon closely linked to enhanced activation of the cGAS–STING pathway and increased tumor immunogenicity described above. Similarly, deficiency in another subunit, ARID1B, also potentiates anti-tumor immunity through a comparable mechanism—impairing DNA repair and activating the cGAS–STING pathway—suggesting a conserved role for multiple members of this complex in regulating tumor immunity (Zhu et al., 2024; Bayona-Feliu et al., 2021). This phenomenon reveals the double-edged sword effect of aberrant chromatin remodeling and provides a new theoretical basis for strategies based on synthetic lethality and combination immunotherapy.

Moreover, nuanced regulation at the level of nucleosome positioning directly impacts the ultimate output of the cGAS–STING pathway. Histone chaperones and chromatin remodelers precisely regulate nucleosome occupancy and positioning at key interferon-stimulated gene loci, such as IFNB1, thereby controlling the accessibility of transcription factors such as IRF3 and NF-κB to target gene promoters. In tumor cells, these mechanisms are often dysregulated, leading to altered expression profiles of interferon-stimulated genes, which ultimately affect the intensity and persistence of the anti-tumor immune response. This dynamic regulation at the nucleosome level constitutes a critical determinant of the response fidelity of the cGAS–STING pathway.

In summary, tumor cells employ a multi-layered network of epigenetic mechanisms, including DNA methylation, histone modifications, and chromatin remodeling, to suppress the activity of the cGAS–STING pathway. This epigenetic silencing not only helps tumor cells evade immune clearance during evolution but also represents a key factor contributing to resistance against current immunotherapies. Notably, the epigenetic state of the pathway is not static; its dynamic changes may even confer new survival advantages upon tumor cells. For instance, studies have indicated that under the pressure of cancer therapy, the activation of the intrinsic cGAS–STING response within tumor cells (distinct from its function in immune cells) may paradoxically help them acquire and maintain drug resistance, revealing another facet of this pathway’s role in tumor cell-autonomous regulation (Lv et al., 2023). Therefore, a profound understanding of these complex epigenetic regulatory mechanisms and the use of epigenetic drugs (such as DNA methyltransferase inhibitors, HDAC inhibitors, and EZH2 inhibitors) to reverse pathway silencing has become a crucial breakthrough for overcoming immunotherapy resistance and enhancing the efficacy of existing therapies. Integrating multi-target intervention strategies that target the epigenetic regulatory network holds promise for propelling tumor immunotherapy into a new era of enhanced precision and synergistic combination.

### 4.3 Regulation at the protein level

Post-translational modifications (PTMs) constitute a sophisticated molecular switch network that critically governs the intensity and duration of cGAS–STING signaling. Tumor cells frequently hijack or disrupt these modification mechanisms to evade immune surveillance and sculpt a tumor microenvironment conducive to their growth (Shen et al., 2025; Shim et al., 2025). Consequently, a profound understanding of the functional roles of PTMs in this pathway is essential for elucidating the mechanisms of tumor immune evasion.

Ubiquitination serves as a core regulatory mechanism modulating cGAS–STING pathway activity, primarily dictating the stability of key signaling components through proteasomal degradation. Various E3 ubiquitin ligases target cGAS or STING, catalyzing the attachment of K48-linked polyubiquitin chains that mark them for proteasomal degradation, thereby negatively regulating the pathway. For instance, TRIM27, highly expressed in ovarian cancer, promotes the K48-linked ubiquitination and degradation of AMPKα, subsequently suppressing AMPK signaling and inactivating the cGAS–STING pathway, which in turn fuels tumor progression (Wang L. et al., 2025). In MSS gastric cancer, TRIM6 catalyzes K27-linked polyubiquitination of cGAS, targeting it for degradation. This inhibition of cGAS–STING-mediated antitumor immunity contributes to resistance against immune checkpoint inhibitor therapy (Niu et al., 2025). Conversely, deubiquitinating enzymes such as USP29 remove ubiquitin chains from cGAS, stabilizing its protein levels and thereby enhancing STING signaling. This dynamic equilibrium between ubiquitination and deubiquitination provides the foundation for maintaining pathway homeostasis and enabling rapid responses under stress conditions. Thus, dysregulation of ubiquitination represents a fundamental molecular basis for immune evasion by tumor cells.

Reversible phosphorylation similarly provides critical regulatory switches for the cGAS–STING pathway. On one hand, kinase-mediated phosphorylation can suppress pathway activity, forming negative feedback loops. For example, in environments rich in growth factor signaling, AKT kinase-mediated phosphorylation of cGAS at Ser291 inhibits its enzymatic activity, representing a vital mechanism for preventing aberrant cGAS activation (Wang et al., 2024b). Additionally, ULK1 kinase-mediated phosphorylation of STING at Ser366 promotes its autophagic degradation, facilitating the timely termination of immune responses following signal transduction. On the other hand, TBK1-mediated phosphorylation of STING is essential for its activation, IRF3 recruitment, and initiation of downstream signaling. Tumor cells can manipulate the activity of these kinases and phosphatases to alter cGAS–STING pathway output. For instance, in HER2^+^ breast cancer exhibiting Herceptin resistance, a positive feedback loop has been observed involving AKT hyperactivation and concomitant suppression of the cGAS–STING pathway (Zhang et al., 2024a). These findings underscore the pivotal role of dynamic phosphorylation changes in maintaining immune homeostasis and facilitating tumor immune evasion.

SUMOylation represents another important layer of regulation for the cGAS–STING pathway. Research indicates that STING SUMOylation, mediated by SUMO1, can inhibit its palmitoylation and subsequent activation at the Golgi apparatus, thereby dampening signal strength (Li et al., 2025). PIAS family proteins, functioning as SUMO E3 ligases, promote STING SUMOylation and negatively regulate antiviral immune responses. Interestingly, in pancreatic cancer models, mild microwave hyperthermia was found to relieve this inhibition by reducing SUMO1 expression, leading to a time-delayed activation of the cGAS–STING pathway and synergistic effects with immune checkpoint blockade therapy (Li et al., 2025). This suggests that aberrant expression or activity of SUMOylation-related enzymes in tumors may contribute to functional silencing of the cGAS–STING pathway and that targeting this modification process could potentially reverse this state. Consequently, SUMOylation is gaining increasing attention as a potential interventional target.

Palmitoylation is an activating modification essential for STING translocation from the endoplasmic reticulum to the Golgi apparatus and the formation of functional signaling complexes. The palmitoyl acyltransferases ZDHHC3 and ZDHHC7 mediate palmitoylation of STING at Cys88 and Cys91, promoting its clustering at the Golgi and recruitment of downstream signaling molecules. The palmitoylation inhibitor 2-bromopalmitate effectively blocks STING activation. Certain tumor cells may suppress STING signaling by downregulating the expression of these palmitoyltransferases. Furthermore, studies have revealed that endogenous metabolites, such as nitro-fatty acids, can competitively inhibit STING palmitoylation, establishing a novel molecular link for metabolic–immune crosstalk. Concurrently, radiotherapy-induced increases in cholesterol synthesis have been found to inhibit STING palmitoylation and subsequent activation by sequestering STING at the endoplasmic reticulum, unveiling a new mechanism whereby tumor metabolic reprogramming facilitates immune evasion (Zhu L. et al., 2025). These insights not only deepen our understanding of the interplay between metabolism and immunity but also provide a theoretical foundation for developing novel combination therapeutic strategies.

In summary, the cGAS–STING pathway is meticulously regulated by a diverse array of post-translational modifications. These modifications constitute a complex regulatory network ensuring the timely initiation and termination of immune responses. Tumor cells disrupt the balance of this network through aberrant expression or activation of specific modifying enzymes (e.g., E3 ligases and kinases) or by exploiting metabolic byproducts, thereby suppressing the antitumor immune functions of the cGAS–STING pathway and achieving immune evasion. A thorough understanding of these regulatory mechanisms at the protein level is instrumental not only for clarifying new principles of tumor immune evasion but also for providing a robust theoretical foundation for the development of targeted therapeutic strategies, such as combining STING agonists with inhibitors of specific modifying enzymes.

### 4.4 Bidirectional regulation of autophagy and the cGAS–STING pathway

Autophagy, a highly conserved intracellular degradation system, is primarily responsible for clearing damaged organelles and protein aggregates. In recent years, it has also been shown to engage in a complex bidirectional regulatory relationship with the cGAS–STING pathway. This interaction is crucial for maintaining immune homeostasis, preventing excessive inflammation, and modulating the tumor immune microenvironment (Schmid et al., 2024; Lu et al., 2023). On one hand, autophagy constrains cGAS–STING signaling through several converging mechanisms, including the clearance of cytosolic DNA ligands and the direct degradation of activated pathway components. On the other hand, activation of the cGAS–STING pathway can itself induce autophagy, forming a negative feedback loop that ensures the appropriateness and controllability of immune responses. Therefore, a thorough dissection of the interplay between autophagy and the cGAS–STING pathway is of great significance for understanding the maintenance of immune homeostasis and for intervening in related diseases.

The inhibitory effect of autophagy on the cGAS–STING pathway is exerted at multiple levels, including the clearance of cytosolic DNA and the direct degradation of key pathway components. First, the autophagy receptor p62 can recognize and bind double-stranded DNA in the cytoplasm, targeting it for degradation via the autophagolysosomal pathway, thereby reducing the availability of ligands recognized by cGAS. Furthermore, the autophagy-related protein Beclin1 has been found to facilitate the interaction between cGAS and p62, thereby enhancing the selective autophagic degradation of cGAS itself. Critically, mitophagy, by clearing damaged or dysfunctional mitochondria and preventing the release of mtDNA, is considered one of the core mechanisms controlling cGAS–STING activation (Wang Y. et al., 2026). Deficiencies in PINK1–Parkin-mediated mitophagy lead to the accumulation of damaged mitochondria, which, in turn, prompts the leakage of mtDNA into the cytoplasm and persistently activates the cGAS–STING pathway, closely associated with the chronic inflammatory phenotypes observed in neurodegenerative diseases such as Parkinson’s disease. Consequently, through its multi-layered fine-tuning, autophagy constitutes a critical barrier that restricts overactivation of the cGAS–STING pathway.

Notably, upon activation, STING can also directly induce autophagy in a manner independent of its classical downstream TBK1–IRF3 signaling axis. After translocating from the ER to the ER-Golgi intermediate compartment, the C-terminal tail of activated STING can directly interact with the key autophagy protein LC3, thereby recruiting the autophagic machinery and forming autophagosomes containing STING and cytosolic DNA (Yan et al., 2022). This non-canonical induction of autophagy, which does not depend on classical autophagy initiation factors such as ULK1 and Beclin1, represents a novel cellular response mechanism directly triggered by innate immune signaling and constitutes a negative feedback loop following cGAS–STING pathway activation. The discovery of this non-canonical autophagy unveils a more direct mode of crosstalk between innate immunity and cellular homeostasis.

During tumor development and progression, cancer cells often hijack the immunomodulatory function of autophagy to achieve immune escape. By upregulating basal autophagy, tumor cells can more efficiently clear spontaneously generated cytosolic DNA and activate STING, thereby dampening cGAS–STING signaling, reducing tumor immunogenicity, and enhancing survival under immune surveillance. Studies have shown that during chemotherapy- or radiotherapy-induced tumor cell death, pharmacological inhibition of autophagy can significantly enhance the accumulation of cytosolic DNA, consequently potentiating the activation of the cGAS–STING pathway and downstream anti-tumor immune responses (Zhao et al., 2022). Therefore, combining autophagy inhibitors with immunotherapeutic strategies, such as immune checkpoint blockade, has emerged as a promising therapeutic approach to releasing the “brake” imposed by tumor cells on the cGAS–STING pathway and re-initiating the anti-tumor immunity cycle. This discovery provides a solid theoretical foundation for the development of tumor immunotherapies based on autophagy modulation.

In summary, the mutual regulation between autophagy and the cGAS–STING pathway exhibits significant tissue and cell-type specificity. In normal immune cells, autophagy helps prevent autoimmune diseases by limiting cGAS–STING hyperactivation. In contrast, within tumor cells, this protective mechanism can be hijacked, evolving into a tool for immune evasion. Clarifying the cell-type- and microenvironment-specific crosstalk between autophagy and the cGAS–STING pathway will therefore be central to designing precise and effective combination therapies. For instance, rationally combining autophagy inhibitors with STING agonists or immune checkpoint inhibitors could maximize the body’s anti-tumor immune response. In the future, precision intervention strategies targeting this regulatory network hold promise for achieving breakthroughs in cancer immunotherapy.

### 4.5 Impact of the tumor microenvironment on cGAS–STING

The tumor microenvironment is a highly heterogeneous, dynamically evolving complex system characterized by hypoxia, metabolic reprogramming, acidosis, and a specialized niche comprising diverse stromal cells and the ECM (Lu et al., 2021; Zhang et al., 2024e; Qi et al., 2025; Zhang B. et al., 2025). Rather than operating in isolation, these factors engage in complex signaling networks to exert multi-faceted regulation of cGAS–STING pathway activity in both tumor and immune cells. Consequently, this profoundly shapes the ultimate functional output of the pathway—determining whether it ignites potent antitumor immunity or, conversely, fosters tumor immune evasion and progression.

#### 4.5.1 Hypoxia

Hypoxia is a prevalent feature of solid tumors and exerts a dual, concentration- and time-dependent effect on the cGAS–STING pathway. On one hand, the hypoxia-inducible factor HIF-1α can directly bind to the promoter region of the STING gene, upregulating its transcription and thereby priming the pathway with increased signaling molecules (Xiang et al., 2023). Conversely, HIF-1α also concurrently induces the expression of immunosuppressive molecules such as PD-L1 and VEGF. This critically alters the functional outcome of STING activation, steering it from promoting inflammation toward sculpting an immunosuppressive microenvironment. More importantly, hypoxia-induced mitochondrial dysfunction serves as a pivotal regulatory node. Hypoxic stress triggers the opening of the mitochondrial permeability transition pore (mPTP), facilitating the release of mtDNA into the cytosol, where it acts as a potent endogenous danger-associated molecular pattern to activate the cGAS–STING pathway (Xia et al., 2025). Simultaneously, however, hypoxia also induces autophagy and upregulates the expression of the DNA exonuclease TREX1, thereby promoting the clearance of cytosolic dsDNA and activated signaling components and negatively regulating signaling strength to prevent excessive inflammatory damage. This intricate interplay results in a context-dependent shift under chronic hypoxia, whereby overall function of the cGAS–STING pathway may transition from transient immune activation to sustained pro-tumorigenic activities, including the promotion of tumor cell survival, EMT, and immune evasion.

#### 4.5.2 Metabolic reprogramming

Metabolic reprogramming in tumor cells not only fulfills their elevated energetic and biosynthetic demands for rapid proliferation but also regulates the cGAS–STING pathway through multiple mechanisms, constituting a novel strategy for tumor immune evasion. Cholesterol metabolism represents a critical node in this process. Research indicates that radiotherapy can induce tumor cells to enhance cholesterol synthesis via the HMGCR pathway. Newly synthesized cholesterol can directly bind to the STING protein, retaining it in the endoplasmic reticulum and thereby inhibiting its translocation to the Golgi apparatus and subsequent activation—a significant mechanism contributing to radiotherapy resistance (Zhu L. et al., 2025). Furthermore, FAO metabolism is involved in regulating immune cell function. For instance, in TAMs, the FAO metabolic state influences the intensity of STING signaling and the expression of SIRPα, thereby modulating their phagocytic capacity (Li Y. et al., 2024). Similarly, the lactate accumulation resulting from enhanced glycolysis not only inhibits cGAS enzymatic activity—by acidifying the cytoplasmic environment and reducing its DNA-binding affinity and catalytic efficiency—but also can act as a signaling molecule, directly suppressing immune cell function via the GPR81 receptor and indirectly dampening the anti-tumor immunity mediated by the cGAS–STING pathway.

#### 4.5.3 Acidosis

Acidosis within the tumor microenvironment, a direct consequence of metabolic reprogramming, exerts a dual modulatory effect on the cGAS–STING pathway. A moderately acidic environment (approximately pH 6.5–6.8) can act as a cellular stressor, inducing autophagy. This process facilitates the clearance of leaked cytosolic dsDNA and activated STING protein complexes, thereby serving as a negative feedback mechanism to limit signal amplification. However, when acidosis becomes more severe, the excessively acidic environment directly inhibits cGAS enzymatic activity. It reduces the binding affinity of cGAS for dsDNA and its efficiency in catalyzing cGAMP synthesis, thereby effectively blocking pathway activation. Therefore, the degree of acidosis dictates whether it exerts a mild regulatory or a potent inhibitory effect on the cGAS–STING pathway.

#### 4.5.4 Regulation by stromal cells and the extracellular matrix

Stromal cells within the tumor microenvironment, particularly cancer-associated fibroblasts (CAFs), profoundly influence the cGAS–STING pathway through diverse mechanisms. Activated CAFs not only secrete soluble factors such as TGF-β and IL-6, which alter the expression levels of cGAS–STING pathway components in both tumor and immune cells (Kanoda et al., 2025), but, more importantly, they also remodel the ECM. This remodeling creates a physical barrier that directly impedes the infiltration of cytotoxic T lymphocytes (CTLs) and fosters an immunosuppressive milieu. Research has directly demonstrated that specific CAF subsets can downregulate the expression of cGAS and STING in colorectal cancer cells, suggesting this as a key mechanism by which tumor cells acquire an immune evasion phenotype under the influence of CAFs (Kanoda et al., 2025). Furthermore, ECM components themselves are active participants in this regulatory network. For instance, hyaluronan fragments can be recognized by TLR2/4, activating the NF-κB pathway and synergizing with STING downstream signaling to amplify inflammatory responses. Conversely, an intact basement membrane restricts the penetration of immune cells and STING agonists, thereby reducing the efficiency of therapeutic delivery and compromising the efficacy of cGAS–STING-based immunotherapies (Xia et al., 2025). These findings underscore that targeting CAFs or remodeling the ECM to dismantle both the “physical” and “functional” barriers they impose represents a crucial strategy to enhance the therapeutic benefits of cGAS–STING agonists.

## 5 Therapeutic strategies targeting the cGAS–STING pathway

**FIGURE 4:**
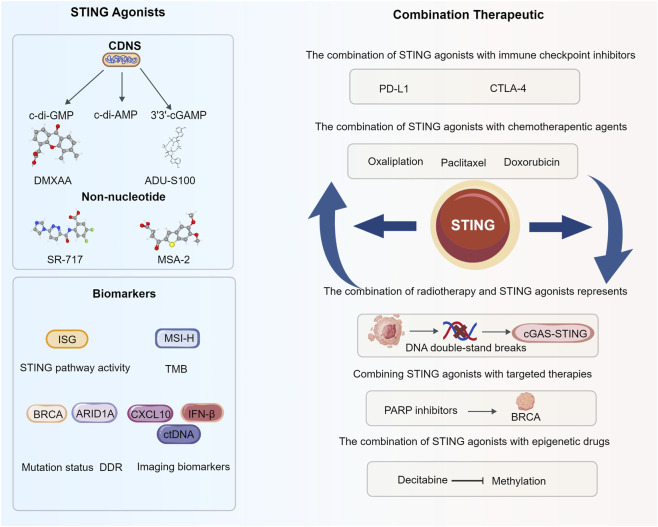
Therapeutic strategies and clinical prospects targeting the cGAS–STING pathway. This figure summarizes current therapeutic strategies and biomarker frameworks targeting the cGAS–STING pathway. (Left upper, STING agonists) Agonists span two structural classes: cyclic dinucleotides (CDNs), including natural ligands (c-di-GMP, c-di-AMP, and 3′3′-cGAMP) and synthetic analogs (DMXAA and ADU-S100); and non-nucleotide small molecules with improved pharmacokinetic properties (SR-717 and MSA-2). (Left lower, biomarkers) Key biomarkers for patient selection and efficacy monitoring include STING pathway activity markers (ISG signatures), microsatellite instability (MSI-H), tumor mutational burden (TMB), DNA damage repair gene mutation status (BRCA and ARID1A), and circulating biomarkers (CXCL10, IFN-β, and ctDNA). (Right, combination therapeutic strategies) STING agonists are being evaluated in combination with immune checkpoint inhibitors (PD-L1 and CTLA-4); chemotherapeutic agents (oxaliplatin, paclitaxel, and doxorubicin); radiotherapy, which induces DNA double-strand breaks activating cGAS–STING; targeted therapies (PARP inhibitors targeting BRCA-mutant tumors); and epigenetic drugs (decitabine, inhibiting DNA methylation).

Targeted modulation of the cGAS–STING pathway has emerged as a core strategy in cancer immunotherapy, with the primary objective of converting immunologically “cold” tumors into “hot” tumors to elicit a robust endogenous anti-tumor immune response (Wang et al., 2020). Based on their chemical structures and mechanisms of action, existing modulators can be systematically classified into three principal categories: STING agonists, STING antagonists, and cGAS inhibitors (Figure 4; Table 2).

**TABLE 2:** cGAS–STING pathway modulators: classification, mechanisms, and clinical status.

Category	Class	Agent	Clinical status	Mechanism and key features
STING agonists	CDN	ADU-S100 (MIW815)	Phase I/II completed (NCT02675439, NCT03172936)	Phosphorothioate-modified to enhance stability; activates all human STING variants (Samson and Ablasser, 2022)
		MK-1454	Phase I discontinued	Direct STING agonist. No objective responses as monotherapy or with pembrolizumab (Samson and Ablasser, 2022)
		SB11285	Phase I ongoing (NCT04096638)	Direct STING agonist. Acceptable safety; immune activation in some patients
		BMS-986301	Phase I completed (NCT03956680)	Direct STING agonist. Anti-tumor immunity in preclinical models
		E7766	Phase I discontinued	Macrocycle-bridged CDN; pan-genotypic activity
	Non-CDN	diABZI	Preclinical/early clinical	Dimeric compound; high human STING affinity; suitable for intravenous (IV) administration (Ramanjulu et al., 2018)
		TAK-676	Phase I/II ongoing (NCT04420884)	Direct STING agonist. Evaluated with pembrolizumab (Carideo Cunniff et al., 2022)
		MSA-2	Preclinical	Non-nucleotide; high oral bioavailability (Pan et al., 2020)
		SR-717	Preclinical	Rationally designed via crystal structures; functions as a cGAMP mimetic to stabilize STING in the active conformation (Chin et al., 2020)
		SNX281	Phase I terminated (NCT04609579)	Direct STING agonist. Potent systemic activity; favorable PK profile
		KL340399	Phase I completed (NCT05549804)	Direct STING agonist. Evaluated in advanced solid tumors
STING antagonists	Covalent/selective small molecules	H-151	Preclinical	Covalent inhibitor; blocks STING palmitoylation and ER-to-Golgi translocation (Haag et al., 2018)
		C-176	Preclinical	Murine-specific STING inhibitor; used for *in vivo* modeling (Haag et al., 2018)
cGAS inhibitors	Competitive catalytic site inhibitors	RU.521	Preclinical	Competitively binds to the cGAS catalytic site; prevents cGAMP synthesis in chronic inflammation (Vincent et al., 2017)
		PF-06928215	Preclinical	Competitive cGAS active-site inhibitor. Tool compound; basis for next-generation cGAS inhibitor design (Hall et al., 2017)

### 5.1 Evolution of STING agonists: CDNs and non-CDNs

Cyclic dinucleotides (CDNs): As natural ligands, CDNs (e.g., bacterial c-di-GMP, c-di-AMP, and mammalian 2′3′-cGAMP) bind with high affinity to the STING ligand-binding domain (LBD) (Samson and Ablasser, 2022). However, natural CDNs face significant clinical hurdles due to their susceptibility to nuclease degradation, suboptimal pharmacokinetic properties, and low cellular uptake efficiency resulting from their negative charge (polyanionic nature). To overcome these limitations, researchers have developed CDN analogs through chemical modification strategies, such as the introduction of phosphorothioate linkages to enhance enzymatic resistance (Zhou et al., 2023). ADU-S100 (also known as MIW815 or ML-RR-S2 CDA), the first synthetic CDN-based agonist in clinical trials, epitomizes this approach through specific phosphorothioate bond replacement of c-di-AMP. While preclinical studies showed potent responses, subsequent Phase I clinical trials revealed limited efficacy as a monotherapy or in combination with PD-1 inhibitors in advanced solid tumors (Shen et al., 2025; Garland et al., 2022). This outcome suggests that simple chemical modification alone is insufficient to overcome complex *in vivo* barriers, necessitating advanced delivery systems. Notably, MK-1454 has been extensively evaluated in combination with pembrolizumab; SB11285 is currently in Phase I/II clinical trials to assess its safety and immune-activating potential. Furthermore, BMS-986301 and the macrocycle-bridged E7766 further expand the CDN repertoire with improved pan-genotypic STING activation.

Non-nucleotide small molecules: Given the inherent limitations of CDNs, non-nucleotide agonists offer entirely new research directions. The flavonoid compound DMXAA was the first to be discovered to possess significant anti-tumor activity in mice; however, it failed in clinical trials due to critical species-specific structural differences that prevent it from effectively binding to human STING (Zheng et al., 2020). This lesson underscores the critical importance of considering human STING structural features during early development. Subsequent breakthroughs include dimeric amidobenzimidazoles, represented by diABZI, which exhibit an exceptional ability to activate human STING with superior cellular permeability, thereby enabling effective signaling following intravenous administration (Gan et al., 2021; Ramanjulu et al., 2018). TAK-676 (dazostinag), another amidobenzimidazole-based non-CDN STING agonist developed by Takeda, was specifically designed for intravenous systemic delivery and is currently being evaluated in Phase I/II clinical trials (NCT04420884) in combination with pembrolizumab for advanced solid tumors (Carideo Cunniff et al., 2022). A comprehensive review of benzimidazole-derived non-CDN STING agonists, encompassing their structure-activity relationships, mechanisms of action, and therapeutic potential, further highlights this class as a promising platform for next-generation immunotherapy (Pavani et al., 2026a). Additionally, structurally distinct agonists such as SR-717 (rationally designed based on crystal structures) and the orally bioavailable MSA-2 provide robust proof of concept for systemic administration strategies (Chin et al., 2020; Pan et al., 2020). Importantly, next-generation candidates such as CRD5500, which demonstrates broad activity across diverse human STING variants, and clinical candidates SNX281 and KL340399 further expand the repertoire of clinically viable STING-targeted agents.

### 5.2 STING antagonists and cGAS inhibitors: addressing pathway duality

In response to the dichotomous role of the cGAS–STING pathway, whereby chronic activation can foster an immunosuppressive and pro-metastatic environment, researchers are actively developing inhibitors to curb pathological signaling. STING antagonists, such as H-151 and C-176, represent viable strategies for contexts where overactivation drives resistance (Haag et al., 2018). H-151, a covalent STING inhibitor, functions by blocking the palmitoylation of STING, thereby preventing its translocation from the endoplasmic reticulum to the Golgi and terminating downstream signal transduction. Upstream, cGAS inhibitors such as RU.521 and PF-06928215 are being explored to suppress cGAMP-driven inflammation in chronic contexts (Vincent et al., 2017; Hall et al., 2017). This balanced therapeutic framework, spanning from potent agonism to precise inhibition, provides a rational basis for precision oncology targeting the cGAS–STING axis.

### 5.3 Combination therapeutic strategies with STING agonists

Given the central role of the cGAS–STING pathway in bridging innate and adaptive immunity, monotherapy often faces challenges in overcoming the complex immunosuppressive network of tumors and the regulatory intricacies of this pathway. Consequently, combining STING agonists with other therapeutic modalities to achieve synergistic enhancement of anti-tumor immunity and overcome treatment resistance has become a mainstream research direction. This combination strategy holds the potential to reshape the landscape of cancer immunotherapy.

The combination of STING agonists with immune checkpoint inhibitors (ICIs) is currently the most extensively studied strategy with the greatest potential for clinical translation. The rationale lies in their highly complementary mechanisms of action: STING agonists activate innate immunity, promoting dendritic cell maturation and antigen presentation, thereby enhancing tumor immunogenicity and T-cell infiltration into the tumor microenvironment. This effectively converts immunologically “cold” tumors into “hot” tumors, creating a favorable condition for the application of ICIs. Conversely, immune checkpoint inhibitors (such as anti-PD-1/PD-L1 or anti-CTLA-4 antibodies) relieve the inhibition imposed on effector T cells by the tumor microenvironment, restoring their cytotoxic function (Jiang et al., 2020; Tian et al., 2022). This synergistic interaction can more comprehensively activate the cancer-immunity cycle, generating durable and potent anti-tumor immune responses. This combinatorial approach has thus emerged as a promising avenue to overcome the bottlenecks currently limiting the efficacy of immunotherapy.

Numerous preclinical studies have unequivocally demonstrated the remarkable efficacy of such combination regimens. For instance, the combination of the early STING agonist ADU-S100 with an anti-PD-1 antibody not only induced complete tumor regression but also generated long-lasting immunological memory, preventing tumor recurrence in a murine colon cancer model. Similarly, the combination of the novel non-nucleotide STING agonist diABZI with an anti-PD-L1 antibody significantly enhances the efficacy of immunotherapy in triple-negative breast cancer (Butterfield and Najjar, 2024). Importantly, these combination therapies inhibit not only primary tumor growth but also effectively control distant metastatic lesions, an effect largely attributed to the systemic, body-wide anti-tumor immune response induced by STING agonists (Tian et al., 2022; Butterfield and Najjar, 2024). These findings provide a robust scientific foundation for clinical translation.

The combination of STING agonists with chemotherapeutic agents also demonstrates significant potential. The mechanisms of action of several commonly used chemotherapeutic agents, such as oxaliplatin, paclitaxel, doxorubicin, and camptothecin derivatives, involve the induction of DNA damage and cellular stress. This leads to genomic instability and the accumulation of cytosolic DNA, including both nuclear and mitochondrial DNA, thereby endogenously activating the cGAS–STING pathway (Liang et al., 2024; Butterfield and Najjar, 2024; Hu Y. et al., 2021). Combining these agents with STING agonists can further amplify this effect, enhancing immunogenic cell death of tumor cells and activating tumor-specific T-cell responses (Liang et al., 2024). Studies have shown that the combination of oxaliplatin and a STING agonist induced superior anti-tumor immunity compared to either agent alone in colorectal cancer models. Likewise, paclitaxel, which activates cGAS–STING signaling by inducing micronuclei formation in cancer cells, synergizes with STING agonists to significantly enhance CD8^+^ T-cell infiltration and tumor suppression in triple-negative breast cancer (Hu Y. et al., 2021). This combination strategy offers a promising new approach to enhancing the immune effects of conventional chemotherapy.

The combination of radiotherapy and STING agonists represents an ideal strategy built upon the concept of the “abscopal effect.” Radiotherapy activates the cGAS–STING pathway by inducing DNA double-strand breaks in tumor cells, leading to chromosomal mis-segregation, micronucleus formation, and the subsequent release of DNA into the cytoplasm (Xue et al., 2022; Storozynsky and Hitt, 2020). However, the immune activation induced by radiotherapy alone is often insufficient to overcome the immunosuppressive tumor microenvironment. STING agonists, functioning as potent immune adjuvants, can significantly amplify the immunostimulatory effects of radiotherapy. They promote dendritic cell activation and cross-presentation, thereby enhancing effector T-cell infiltration into the tumor and their cytotoxic function. Preclinical studies have demonstrated that combining radiotherapy with STING agonists markedly enhances the abscopal effect in murine models of lung and colorectal cancer, effectively inhibiting the growth of unirradiated metastatic lesions (Butterfield and Najjar, 2024; Yang et al., 2023). This provides a novel strategy to overcome radiotherapy resistance and achieve profound synergy between radiotherapy and immunotherapy, a combination that is progressively moving from concept toward clinical validation.

Combining STING agonists with targeted therapies is also gaining increasing attention, particularly concerning targets within DNA damage repair pathways. For instance, PARP inhibitors, used in tumors with homologous recombination repair deficiencies such as BRCA mutations, activate the cGAS–STING pathway by inducing persistent DNA damage and accumulation of cytosolic DNA (Wang T. et al., 2023). However, the immune activation resulting from PARP inhibitor monotherapy is limited, and combining it with a STING agonist can synergistically enhance this effect. Furthermore, inhibitors of ATM or ATR exacerbate chemotherapy- and radiotherapy-induced DNA damage and cytosolic DNA accumulation by interfering with key signaling pathways involved in DNA damage repair. Their combination with STING agonists has demonstrated synergistic anti-tumor activity in various tumor models (Hu M. et al., 2021; Taniguchi et al., 2024). Similarly, CDK4/6 inhibitors, while inducing cell cycle arrest, can also trigger a senescence-associated secretory phenotype; combining them with STING agonists further potentiates anti-tumor immunity (Wang T. et al., 2023). This direction holds promise for expanding the immune-synergistic applications of targeted drugs.

The combination of STING agonists with epigenetic drugs is an emerging therapeutic strategy aimed at restoring or enhancing tumor cell sensitivity to STING signaling through epigenetic modulation. Tumor cells often evade immune surveillance by epigenetically silencing cGAS or STING expression, for example, through promoter methylation. DNA methyltransferase inhibitors, such as decitabine, can reverse STING promoter methylation, restore STING expression, and consequently sensitize tumor cells to STING agonists (Sasaki et al., 2023). HDAC inhibitors, by relaxing chromatin structure, facilitate the transcription of interferon-stimulated genes and synergize with STING agonists to enhance type I interferon responses (Zhang et al., 2024b). Moreover, EZH2 inhibitors, by removing the repressive H3K27me3 mark, can also upregulate STING expression and augment the efficacy of combination therapy. These findings offer novel insights for epigenetic immunotherapy.

Finally, the application of STING agonists as immune adjuvants in combination with tumor vaccines holds substantial promise. STING agonists are natural immune adjuvants that effectively promote the uptake, processing, and presentation of antigens by dendritic cells, while also inducing their maturation and migration. This significantly enhances the vaccine-induced tumor-specific T-cell response (Zhang et al., 2024b). Preclinical studies have confirmed that combining STING agonists with peptide vaccines or tumor cell lysate vaccines induces more potent anti-tumor immunity in murine models. Based on this solid theoretical foundation and robust preclinical data, numerous clinical trials are currently evaluating the combination of STING agonists with various therapies, including immune checkpoint inhibitors, chemotherapy, radiotherapy, and personalized cancer vaccines. The prospects for their clinical translation are highly promising. This mechanism-guided combination strategy is propelling cancer immunotherapy toward a more precise and effective future.

### 5.4 Application of nanodelivery systems in cGAS–STING-targeted therapies

**FIGURE 5:**
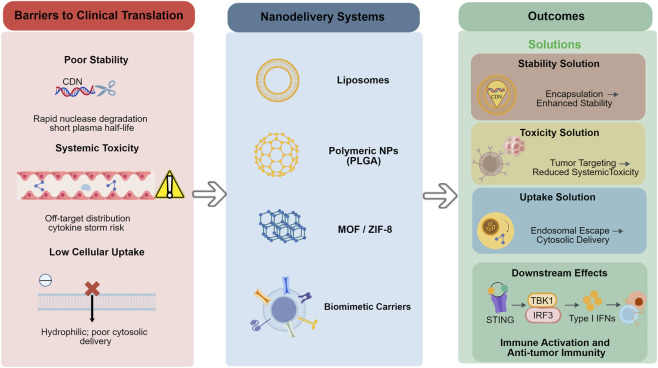
Nanocarrier-based delivery strategies for STING agonists: challenges and solutions. This figure illustrates the principal barriers to clinical translation of STING agonists and how nanocarrier-based delivery systems address them. (Left panel) Three key pharmacological barriers are depicted. (1) Poor stability—CDN-based agonists are susceptible to rapid nuclease degradation and exhibit short plasma half-lives. (2) Systemic toxicity—off-target distribution risks a cytokine storm and organ toxicity. (3) Low cellular uptake—the highly charged, hydrophilic nature of CDNs hinders cytosolic delivery. (Middle panel) Four principal classes of nanodelivery systems are shown: liposomes, polymeric nanoparticles (PLGA), metal-organic frameworks (MOF/ZIF-8), and biomimetic carriers. (Right panel, upper) Corresponding solutions: encapsulation enhances stability; tumor-targeted delivery reduces systemic toxicity; and endosomal escape enables efficient cytosolic release. (Right panel, lower) Successful intracellular delivery activates the STING–TBK1/IRF3 signaling axis, driving type I interferon production and anti-tumor immunity.

Despite the considerable potential of strategies targeting the cGAS–STING pathway in tumor immunotherapy, the clinical translation of STING agonists faces multiple challenges (Figure 5). These include 1) poor stability, as agonists represented by CDNs are susceptible to degradation by nucleases; 2) suboptimal pharmacokinetic properties, characterized by low molecular weight leading to rapid clearance and poor tumor accumulation; 3) delivery barriers, resulting in inefficient cell membrane penetration and endosomal escape required to reach cytosolic targets; and 4) safety risks, where systemic administration may cause non-specific immune activation and “off-target” toxicity (Zhou et al., 2023; Lu et al., 2024b). Consequently, the development of safe and efficient delivery systems is critical for realizing the clinical potential of STING agonists. Ideal nanodelivery systems should possess capabilities such as tumor targeting, controlled release, enhanced bioavailability, and reduced systemic toxicity (Zhou et al., 2023). In recent years, various nanoplatforms have been developed for the precise delivery of STING agonists, achieving significant progress (Peng et al., 2025). This context establishes a crucial foundation for the subsequent design of diverse delivery systems.

Liposomes were among the first nanocarriers employed for STING agonist delivery. Encapsulation within liposomes can effectively protect CDNs from enzymatic degradation *in vivo*, prolong their circulation time, and facilitate passive tumor targeting via the enhanced permeability and retention effect. Cationic liposomes exhibit high encapsulation efficiency for negatively charged CDNs through electrostatic interactions; however, their potential for non-specific toxicity limits clinical application. To overcome this limitation, researchers have developed pH-sensitive liposomes that trigger drug release specifically within the acidic tumor microenvironment, enabling spatially controlled and precise delivery. Studies have shown that liposome-encapsulated c-di-GMP significantly enhances STING activation and anti-tumor immune responses in murine melanoma models. Furthermore, liposomes can be utilized for the co-delivery of STING agonists with other immunomodulators, such as TLR agonists, to achieve synergistic immune activation (Zhou et al., 2023). These advances indicate that liposomes remain a highly practical platform for STING agonist delivery.

Polymeric nanoparticles represent another crucial class of delivery systems, garnering significant attention due to their tunable structures and functional versatility. Poly(lactic-co-glycolic acid) (PLGA) is widely used in STING agonist delivery research owing to its excellent biocompatibility and degradability (Wang T. et al., 2023). PLGA nanoparticles can encapsulate both hydrophilic and hydrophobic agonists, and their drug release kinetics can be precisely modulated by adjusting polymer composition and molecular weight. Cationic polymers, such as polyethyleneimine, can form polyelectrolyte complexes with CDNs via electrostatic interactions, effectively promoting cellular uptake and endosomal escape; however, their potential toxicity requires careful evaluation. Recently, amphiphilic copolymers based on PEG-PLGA have been employed to construct cGAMP nanomicelles, which markedly enhanced STING activation and anti-tumor immunity in mouse tumor models. Additionally, more sophisticated polymer nanostructures, including polymersomes and polymer capsules, are progressively being developed for the targeted delivery of STING agonists and for combination therapies (Peng et al., 2025). These multifunctional polymeric systems offer expanded possibilities for the refined implementation of immunomodulatory strategies.

Inorganic nanomaterials offer new opportunities for STING agonist delivery by leveraging their unique physicochemical properties. Gold nanoparticles can be surface-functionalized with STING agonists, enabling synergistic effects between photothermal therapy and immunotherapy (Zhang W. et al., 2025). Mesoporous silica nanoparticles, characterized by their high surface area and tunable pore size, efficiently load CDNs and facilitate responsive drug release (Zhong et al., 2025). Iron oxide nanoparticles serve a dual function as drug carriers and agents for magnetic resonance imaging-guided therapy. Notably, certain inorganic nanomaterials possess an intrinsic ability to activate the cGAS–STING pathway, allowing for the integration of carrier and agonist functions. For instance, manganese-based nanomaterials release Mn^2+^ ions, which directly sensitize cGAS to dsDNA recognition and synergistically amplify pathway activation (Zhong et al., 2025; Zhang X. et al., 2025; Ma et al., 2025). This strategy of “metallo-immunotherapy,” which harnesses the inherent immune activity of metal ions, opens new avenues for the design of innovative nano-immunotherapies.

Metal-organic frameworks (MOFs) have recently emerged as highly promising multifunctional delivery platforms. These materials, formed through the self-assembly of metal ions and organic ligands, boast ultra-high surface areas, adjustable pore sizes, and favorable biodegradability (Sun et al., 2024a; Zhu S. et al., 2025). Zeolitic imidazolate frameworks (e.g., ZIF-8) can efficiently encapsulate CDNs and degrade within the acidic tumor microenvironment, thereby releasing their payload. More critically, metal ions released from certain MOFs, such as Zn^2+^ and Mn^2+^, can synergize with agonists to co-activate the cGAS–STING pathway. Research demonstrates that Mn-MOF nanoparticles loaded with cGAMP induce potent anti-tumor immunity in a mouse model of breast cancer, an effect further enhanced by combination with anti-PD-L1 therapy (Sun et al., 2024a). Furthermore, MOF-based nanoplatforms can integrate with diverse therapeutic strategies, such as combining with the chemotherapeutic drug mitoxantrone to induce immunogenic cell death, thereby further amplifying STING pathway activation (Zhang et al., 2024f). These findings underscore the significant potential of MOFs in STING agonist delivery and combination immunotherapy.

Biomimetic nano-delivery systems, which utilize cell membranes or vesicles for targeted delivery and immune modulation, have garnered significant attention due to their high biocompatibility and natural targeting capabilities. Nanoparticles coated with tumor cell membranes enable homotypic targeting, enhancing accumulation in tumor tissues (Chen et al., 2023). Engineered bacterial outer membrane vesicles, containing pathogen-associated molecular patterns, can co-activate multiple innate immune pathways, thereby augmenting the immunostimulatory effect (Liang et al., 2025). Studies have shown that bacterial outer membrane vesicles expressing cGAS can generate cGAMP *in situ* within the tumor microenvironment, leading to sustained activation of STING (Liang et al., 2025). Vesicles derived from red blood cells or immune cells also represent promising carriers for STING agonist delivery, given their natural origin and excellent biocompatibility. Additionally, biomimetic systems coated with hybrid cell membranes (e.g., tumor–fibroblast fusion membranes) can remodel the tumor immune microenvironment through multiple mechanisms, resulting in more potent anti-tumor immune responses (Chen et al., 2023). These biomimetic strategies offer new approaches to enhance the targeting and safety of STING agonist delivery.

Stimuli-responsive delivery systems enable spatiotemporally controlled drug release, thereby further improving therapeutic precision. pH-responsive systems, which exploit the acidic tumor microenvironment to trigger drug release, represent one of the most mature delivery strategies. Redox-responsive systems leverage the high intracellular glutathione levels in tumor cells to cleave disulfide bonds and release their payload. Enzyme-responsive systems achieve specific drug release through enzymes highly expressed in the tumor microenvironment, such as matrix metalloproteinases. Photothermal-responsive systems utilize near-infrared light irradiation to induce local heating, which can both trigger drug release and synergize with photothermal therapy to further enhance immunogenic cell death (Feng et al., 2025; Zhou et al., 2025). For instance, one study developed a nucleus-targeted nanoplatform combining photodynamic therapy and chemotherapy, which precisely induced DNA damage upon near-infrared light irradiation, thereby potently activating the cGAS–STING pathway for bladder cancer immunotherapy (Feng et al., 2025). Such intelligent delivery systems provide powerful tools for the precise, controlled release of STING agonists and for combination therapies.

In summary, the diverse landscape of nano-delivery systems is continuously advancing the clinical translation of STING agonists. Future research will focus on developing increasingly intelligent and multifunctional nanoplatforms that integrate targeted delivery, multifaceted immune activation, and combination therapeutic strategies. This integrated approach aims to maximize the potential of the cGAS–STING pathway in tumor immunotherapy and ultimately improve clinical outcomes for patients.

### 5.5 Other targeting strategies

Beyond the direct agonism of STING, researchers are actively exploring the therapeutic potential of targeting other key nodes within the cGAS–STING pathway. This approach aims to achieve a more nuanced regulation of the pathway, tailored to the specific demands of the tumor microenvironment (Samson and Ablasser, 2022; Shen et al., 2025). These multifaceted strategies are designed to overcome the potential challenges associated with STING agonist monotherapy and to offer new avenues for combination treatments.

cGAS inhibitors were initially developed primarily for autoimmune diseases driven by aberrant cGAS activation. However, recent studies have illuminated their potential utility in oncology (Zhou et al., 2023; Chen W. J. et al., 2021). Given the dual role of the cGAS–STING pathway in cancer—particularly the observation that chronic STING activation in certain tumors can promote progression and induce immunosuppression—moderate inhibition of cGAS activity emerges as a potentially beneficial therapeutic strategy (Kwon and Bakhoum, 2020; Zheng et al., 2020). For instance, in lung cancer, persistent STING pathway activation driven by chromosomal instability (CIN) or specific gene mutations (e.g., those conferring EGFR–TKI resistance) may paradoxically fuel tumor progression and therapeutic resistance by promoting EMT or recruiting immunosuppressive cells (Kwon and Bakhoum, 2020; Yonesaka et al., 2025). By blocking such pro-tumorigenic chronic inflammation, cGAS inhibitors hold the potential to remodel the tumor immune microenvironment. Representative cGAS inhibitors, such as RU.521 and PF-06928215, primarily function by competitively binding to the catalytic site of cGAS, thereby inhibiting its enzymatic activity. Nevertheless, the application of cGAS inhibitors in cancer therapy requires careful consideration; their precise indications, optimal dosing schedules, and combination regimens necessitate further validation through preclinical and clinical investigations (Sasaki et al., 2023). Consequently, future research must delineate the safety margins and optimal clinical scenarios for their use in oncology.

TBK1 inhibitors target a key signaling molecule downstream of STING and are primarily employed to curb inflammation driven by pathway hyperactivation. However, since TBK1 serves as a convergence point for multiple signaling cascades (such as NF-κB and AKT), its inhibition can elicit complex biological effects (Shen et al., 2025). Amlexanox, a classical TBK1 inhibitor, has demonstrated anti-inflammatory activity in preclinical models, but its clinical utility has been constrained by limited selectivity. To enhance target specificity, novel TBK1 inhibitors such as GSK8612 and Compound II are under active development. In the context of cancer therapy, the role of TBK1 inhibitors may not be to suppress acute anti-tumor immune responses. Instead, in specific settings where chronic STING pathway activation fosters immunosuppression, they could potentially reverse this suppressive state by blocking downstream signals, thereby remodeling the immune microenvironment (Castle et al., 2024). This suggests that their clinical application will depend on precise patient stratification based on the tumor’s immune context.

Inhibition or phosphorylation modulation of IRF3 represents another potential intervention strategy. IRF3 is a critical transcription factor for inducing type I interferons; thus, directly inhibiting IRF3 would likely block the interferon response and potentially impair anti-tumor immunity. This renders the application of this strategy extremely delicate and necessitates a profound understanding of its appropriate context. Recently, studies have identified small molecules capable of fine-tuning IRF3’s transcriptional activity by modulating its phosphorylation or dimerization status. This “tuning,” rather than a complete “shut-off,” offers a novel approach to intervening in STING signaling, potentially suppressing pathological inflammation while preserving essential immune surveillance functions (Vasiyani, 2026). Therefore, the design of IRF3 modulators should prioritize functional regulation over complete inhibition.

ENPP1 inhibitors represent an effective strategy for indirectly enhancing STING signaling. ENPP1 is an ecto-nuclease that hydrolyzes extracellular 2′3′-cGAMP. Inhibiting ENPP1 activity prolongs the half-life of this second messenger, thereby potentiating the intensity and duration of STING signaling (You et al., 2025). Research indicates that ENPP1 is overexpressed in various tumor types and is closely associated with immunosuppression and poor prognosis. Beyond its membrane-bound form, ENPP1 is also carried on tumor-derived exosomes, where it can effectively hydrolyze cytoplasmic cGAMP, thereby inhibiting the cGAS–STING pathway in immune cells and establishing a novel mechanism of tumor immune evasion (An et al., 2024). Currently, multiple small-molecule inhibitors targeting ENPP1 are in preclinical development. Notably, the mechanism of ENPP1 inhibitors may be two-fold: first, by reducing cGAMP degradation to enhance STING activation and, second, by modulating purinergic signaling, which directly affects the function of immune cells within the tumor microenvironment (Zhang et al., 2024f). Combining ENPP1 inhibitors with STING agonists holds promise for synergistically provoking a more potent anti-tumor immune response. This combinatorial approach offers a compelling strategy to augment the efficacy of therapies eliciting the STING pathway.

Targeting metabolic enzymes associated with the cGAS–STING pathway also presents an attractive therapeutic Frontier. Studies have revealed a close interplay between cellular metabolic states and cGAS–STING pathway activity. For example, inhibitors of HMGCR (statins), a key enzyme in the cholesterol synthesis pathway, can enhance STING activation by reducing intracellular cholesterol levels, thereby relieving its inhibitory effect on STING (Zhu L. et al., 2025; Huang et al., 2024). Similarly, modulators of fatty acid oxidation (FAO) may alter the responsiveness of immune cells, such as CD8^+^ T cells, to STING signaling by influencing their metabolic reprogramming. The activation of STING signaling itself is subject to metabolic regulation, with studies showing that STING can regulate protein translation via the PERK–eIF2α pathway, contributing to cellular senescence and organ fibrosis (Zhang D. et al., 2022). These metabolic intervention strategies open up new dimensions for combination therapies. By reshaping the metabolic landscape of the tumor microenvironment, they hold the potential to improve the overall efficacy of STING-targeted therapies (Xiang et al., 2023). This underscores the promising translational potential of integrating metabolic regulation with immune-based interventions.

## 6 Current status and challenges in clinical translation

### 6.1 Advances in clinical trials

In recent years, various STING-targeted agonists have entered clinical trials, with preliminary results now available. Overall, however, the efficacy data have fallen short of preclinical expectations, highlighting significant challenges in clinical translation (Lu et al., 2026).

ADU-S100 (MIW815), the first CDN STING agonist in clinical trials, demonstrated acceptable safety via intratumoral injection in a Phase I trial (NCT02675439), with tumor shrinkage and immune activation biomarkers observed in some patients (Zhou et al., 2018). However, a subsequent Phase I trial combining ADU-S100 with spartalizumab (NCT03172936) yielded an ORR of only approximately 10%, and later Phase II trials in melanoma and head and neck cancer failed to replicate preclinical efficacy. MK-1454, evaluated in combination with pembrolizumab (NCT03010176), showed a modestly higher ORR of approximately 24% in the combination group versus 7% for monotherapy (Zhou et al., 2018), though responses remained confined to a subset of patients. BMS-986301 completed a Phase I trial (NCT03956680) with a favorable safety profile, but formal efficacy data have not been published. Among other CDN agonists, E7766 (NCT04144140) was withdrawn prior to enrollment due to a sponsor business decision; SB11285 (NCT04096638) remains in Phase I evaluation with acceptable safety; and SNX281 (NCT04609579) was terminated. Collectively, these results underscore the limitations of CDN-based STING agonists in the clinical setting (Table 2).

In the non-nucleotide class, TAK-676 (dazostinag) is being evaluated in a Phase I/II study (NCT04420884) as monotherapy or combined with pembrolizumab in advanced solid tumors, including SCCHN and CRC, with a separate completed Phase I study (NCT04879849) examining its combination with pembrolizumab and radiation therapy in NSCLC, TNBC, and SCCHN, though efficacy results remain unpublished.

The clinical underperformance of STING agonists reflects several converging factors. Trial participants are typically heavily pre-treated with functionally exhausted immune systems, for whom pathway activation alone is insufficient to reverse established immunosuppression. STING genetic polymorphisms—across five major haplotypes (WT, HAQ, AQ, Q, and R232H) with differing agonist responsiveness (Zhang L. et al., 2022)—likely contributed to variable patient responses. Finally, the failure to stratify patients by tumor immune phenotype or STING pathway status limited the identification of responsive subpopulations. Future trials must integrate biomarker-guided patient selection with rational combination strategies to overcome these bottlenecks.

### 6.2 Challenges

Despite the considerable promise of the cGAS–STING pathway as a target for anti-tumor immunotherapy, therapeutic strategies targeting this pathway face several formidable challenges in clinical translation and application.

#### 6.2.1 Intrinsic drug limitations and tumor-mediated resistance

The suboptimal pharmacokinetic and biodistribution properties of STING agonists, particularly CDNs, represent a fundamental obstacle to clinical translation. CDN molecules carry a high density of negative charge and are highly hydrophilic, hindering passive diffusion across the lipid bilayer of cell membranes and resulting in poor cellular uptake. Upon systemic administration, CDNs are rapidly degraded by phosphodiesterases and ectonucleotidases—most notably ENPP1, which hydrolyzes extracellular 2′3′-cGAMP—and by ubiquitous nucleases in the blood, leading to an extremely short circulation half-life *in vivo* (Jacoberger-Foissac et al., 2023). Even with intratumoral injection as a local route of administration, drug distribution within the solid tumors is often heterogeneous, making it difficult to achieve effective coverage of all tumor cells. This route of administration, while effective in subcutaneous mouse models, is often impractical for deep-seated or metastatic tumors in patients, limiting the clinical translatability of preclinical findings (Samson and Ablasser, 2022; Pavani et al., 2026b). These inherent pharmacokinetic drawbacks urgently necessitate the development of innovative drug delivery systems to overcome them. Compounding these limitations, tumor cells have developed diverse intrinsic mechanisms to suppress pathway activity, including epigenetic silencing of cGAS or STING genes through DNA methylation or histone modifications, ubiquitin–proteasome-mediated protein degradation, and mutation or downregulation of key pathway molecules (Sasaki et al., 2023; Huang et al., 2024; Marinello et al., 2022). For instance, SCLC cells frequently exhibit impaired pathway function due to loss of STING and/or cGAS expression (Marinello et al., 2022). In tumors in which STING signaling is already compromised, even potent agonists may fail to initiate effective downstream immune responses, underscoring the need to incorporate assessment of STING pathway activity into both preclinical and clinical study designs. Human STING genetic polymorphism further complicates this picture: five major haplotypes (WT, HAQ, AQ, Q, and R232H) differ substantially in their responsiveness to various agonists (Wang W. et al., 2022). Individuals carrying the R232H variant may show markedly reduced or absent responses to specific CDN agonists. Since many preclinical studies relied on mouse models or human cell lines expressing only the reference STING variant, efficacy in heterogeneous patient populations harboring less-responsive haplotypes may have been systematically overestimated (Samson and Ablasser, 2022). This underscores the urgent need to develop pan-STING agonists with broad interallelic activity and to incorporate STING genotyping into patient stratification strategies in future clinical trials.

#### 6.2.2 Immunosuppressive tumor microenvironment and paradoxical signaling consequences

The TME creates multiple barriers that directly attenuate the efficacy of STING agonists. The abundant infiltration of immunosuppressive cells—including regulatory T cells, MDSCs, and M2-type tumor-associated macrophages—can potently counteract the immune activation induced by STING agonists. Cancer-associated fibroblasts further restrict drug penetration and immune cell infiltration through the secretion of extracellular matrix components that form dense physical barriers (Kabashima et al., 2022; Kanoda et al., 2025). Critically, hypoxia—a hallmark of solid tumors—has been shown to directly suppress STING expression and activity, while the acidic pH of the TME may further impair the stability and cellular uptake of CDN-based agonists (Zhou et al., 2025). In immunologically “cold” tumors or those with a highly suppressive microenvironment, STING agonists may therefore need to be combined with TME-remodeling strategies—such as certain chemotherapies, radiotherapies, or agents targeting stromal components—to fully unleash their immune-activating potential. Beyond these microenvironmental barriers, STING activation itself triggers a critical negative feedback loop: while promoting type I IFN production and T-cell priming, it concurrently upregulates PD-L1 expression on tumor cells and immune cells, limiting the duration and magnitude of the anti-tumor response (Schmid et al., 2024; Liu F. R. et al., 2022). This mechanistic coupling between STING activation and adaptive immune resistance explains, at least in part, why STING agonists as monotherapies have shown limited clinical efficacy and highlights the strong rationale for combination with immune checkpoint inhibitors. More fundamentally, emerging evidence indicates that chronic STING activation can paradoxically drive tumor progression through NF-κB-mediated inflammation, maintenance of cancer cell stemness, induction of chemoresistance, and promotion of metastasis via the secretion of pro-invasive cytokines (Kwon and Bakhoum, 2020; Liu F. R. et al., 2022; Lv et al., 2023). The dual nature of STING signaling thus presents a fundamental therapeutic dilemma: achieving sufficient pathway activation to elicit anti-tumor immunity while avoiding the pro-tumorigenic consequences of chronic overstimulation.

#### 6.2.3 Clinical translation barriers and systemic safety concerns

At the clinical level, systemic administration of STING agonists carries significant toxicity risks. As a pivotal hub of innate immunity, excessive STING activation can precipitate a cytokine storm, triggering severe systemic inflammatory responses resembling septic shock (Yue et al., 2025). Completed Phase I trials have documented toxicities including flu-like symptoms, hypotension, elevated cytokine levels, and increased transaminases. Even with intratumoral injection, drug leakage into systemic circulation can cause off-target inflammatory responses. Moreover, chronic or excessive STING activation might disrupt immune tolerance, potentially inducing or exacerbating autoimmune conditions in genetically predisposed individuals. Careful exploration of dosing regimens and rigorous patient monitoring are therefore imperative during clinical development. Trial participants are also typically heavily pre-treated patients with advanced disease and functionally exhausted immune systems, for whom STING pathway activation alone may be insufficient to reverse established immunosuppression. Finally, early-stage clinical trials frequently failed to stratify patients based on tumor immune phenotype (“hot” vs. “cold”) or STING pathway functional status, thereby limiting the identification of responsive subpopulations. Future progress will require integrating biomarker-guided patient selection—incorporating STING haplotype genotyping, tumor immune phenotyping, and circulating ISG signatures—with rational combination strategies to overcome the current bottlenecks in clinical translation.

### 6.3 Exploration of biomarkers

Given the multifaceted challenges still facing the clinical translation of STING agonists, the development of robust biomarkers is critical for enabling precision therapy, optimizing patient selection, monitoring efficacy, and predicting response (Ji et al., 2025; Sun L. et al., 2025). Ideal biomarkers should accurately reflect the tumor’s immune status and its potential responsiveness to STING-targeted therapies, thereby providing a rationale for the development of personalized treatment strategies.

Biomarkers related to STING pathway activity serve as the most direct predictive indicators of pathway status. By assessing the phosphorylation or expression levels of cGAS, STING, and key downstream molecules such as TBK1 and IRF3 in tumor tissue, the baseline activity of the pathway can be effectively evaluated. Studies have demonstrated that in tumors with high STING expression, such as triple-negative breast cancer (TNBC), patients show more significant responses to agonist therapy (Chen et al., 2024; Nishida et al., 2024). Furthermore, the expression signature of interferon-stimulated genes (ISGs) induced by STING activation, including CXCL10, ISG15, and CCL5, is also used as a surrogate marker for pathway functional activity (Yan et al., 2022; Oh et al., 2024). Notably, the expression level of cGAS itself may also hold prognostic and predictive value. For instance, in patients with lung adenocarcinoma, high expression of cGAS, STING, and TBK1 correlates with improved overall survival in those with localized disease, suggesting their potential as prognostic biomarkers for early-stage patients (Raaby Gammelgaard et al., 2021). However, the relationship between cGAS expression and therapeutic efficacy may vary depending on tumor type and the accompanying immune microenvironment. One study indicated that in patients with PD-L1-high non-small cell lung cancer (NSCLC), high cGAS expression was paradoxically associated with shorter progression-free survival on immunotherapy, potentially linked to a TGF-β-mediated immunosuppressive environment (Ozawa et al., 2024). Consequently, the dynamic changes of these markers during treatment and their spatiotemporal heterogeneity within tumor tissues require further validation through large-scale clinical studies to clarify their clinical utility.

Tumor mutational burden (TMB) and genomic instability metrics are closely associated with cGAS–STING pathway activity. Tumors with high TMB typically accumulate more cytosolic DNA due to replication stress and may therefore be more sensitive to STING agonists (Sokač et al., 2022). Specifically, microsatellite instability-high (MSI-H) colorectal cancer, characterized by high immunogenicity and frequently accompanied by high STING expression, is considered a potential priority population for STING agonist therapy (Kunac et al., 2022; Kaneta et al., 2022). Additionally, the chromosomal instability (CIN) index serves as an important measure of a tumor’s intrinsic immunogenicity. Research shows that CIN can induce cGAS–STING signaling activation (Sokač et al., 2022), but persistent CIN may also lead cancer cells to inactivate this pathway through mechanisms such as epigenetic silencing to evade immune surveillance. Of note, in EGFR-mutant NSCLC, CIN can promote EMT via activation of the cGAS–STING pathway, thereby contributing to acquired resistance to EGFR-TKIs (Yonesaka et al., 2025). These genomic markers can be obtained from routine tumor tissue testing and hold promising prospects for clinical application.

Mutation status in DNA damage repair (DDR) genes critically regulates cGAS–STING pathway activity. Tumors with homologous recombination repair deficiencies, such as those harboring *BRCA1/2* mutations, exhibit impaired repair of DNA double-strand breaks, leading to genomic instability and cytosolic DNA accumulation. This may render them more sensitive to STING agonists or combination strategies with PARP inhibitors (Hu M. et al., 2021; Sun et al., 2024b; Patterson-Forti et al., 2023). Similarly, mutations or inhibition of key kinases in the DNA damage response, such as ATM, ATR, and POLQ, have been shown to enhance STING pathway activation and antitumor immunity induced by radiotherapy or chemotherapy (Taniguchi et al., 2024; van Campen et al., 2025; Sun et al., 2024c). Furthermore, mutations in chromatin remodeling complex genes such as ARID1A can enhance cGAS–STING transcriptional activity by altering chromatin accessibility and are associated with favorable responses to immune checkpoint inhibitors (Zhu et al., 2024). Similarly, loss of ARID1B has been found to impair the DNA damage response and activate the cGAS–STING pathway, suggesting its potential as a biomarker for predicting ICI response (Zhu et al., 2024). Other examples include heterozygous loss of PPP2R2A (encoding the PP2A B55α subunit), which activates the cGAS–STING pathway by increasing cytosolic DNA and sensitizes NSCLC cells to PD-L1 blockade, indicating that PPP2R2A loss may serve as a potential biomarker for guiding ICB therapy (Qiu et al., 2026). Deficiencies in PBRM1 are linked to synthetic lethality with PARP and ATR inhibitors and can activate cGAS–STING signaling by inducing micronuclei and R-loops (Chabanon et al., 2021). Therefore, the mutation status of DDR genes can serve as crucial molecular markers for identifying potentially responsive patient populations, providing a basis for personalized treatment.

Immune microenvironment characteristics represent important biomarkers for predicting response to STING agonists. The baseline infiltration level of CD8^+^ T cells in pre-treatment tumor tissue often predicts a better synergistic effect when combining STING agonists with immune checkpoint inhibitors (Kabashima et al., 2022). Additionally, the maturation status and functional activity of DCs within the tumor microenvironment directly influence the efficiency of antigen presentation following STING activation. Notably, the proportion of immunosuppressive cells, such as MDSCs and Tregs, can reflect the intensity of tumor immunosuppression, suggesting a potential need for combination with corresponding inhibitors to overcome resistance (Jacoberger-Foissac et al., 2023). For instance, loss of macrophage Annexin A1 (ANXA1) in pancreatic cancer can enhance efferocytosis and activate the cGAS–STING pathway, remodeling the immune microenvironment and suggesting ANXA1 as a potential new intervention target and efficacy marker (Hou et al., 2024). Furthermore, the immunosuppressive axis formed by T-cell immunoglobulin and mucin domain-containing protein 3 (TIM-3) and its ligand Galectin-9 is also influenced by STING signaling. Research has found that ATM inhibition can upregulate galectin-9 expression in tumor cells via the cGAS–STING–IFNβ pathway, thereby mediating immune escape. Combining ATM inhibition with a galectin-9 antibody significantly enhances antitumor immunity, offering a novel strategy to overcome resistance to PD-1/PD-L1 blockade (Zheng et al., 2023). Concurrently, STING signaling itself regulates TIM-3; for example, the berberine derivative C51 can promote TIM-3 degradation by activating cGAS–STING, thereby inhibiting lung cancer progression (Bao et al., 2026). Other negative regulators, such as NLRC3, which attenuates antitumor immune responses by suppressing the cGAS–STING pathway, may also serve as potential biomarkers for predicting immunotherapy response based on their expression or activity status (Wang Q. et al., 2024). These immune microenvironment biomarkers can be assessed using techniques such as flow cytometry, immunohistochemistry, or single-cell sequencing, providing multidimensional information for efficacy prediction.

Imaging biomarkers offer a novel approach for non-invasive, dynamic monitoring of systemic STING activation status. For example, the development of PET tracers targeting STING or its ligands enables *in vivo* imaging of STING distribution and activity (Zhou et al., 2023). Additionally, detecting metabolic changes associated with immune activation using magnetic resonance spectroscopy (MRS) could indirectly reflect STING pathway activation. Although the majority of these imaging modalities are still in the preclinical development phase, they have the potential to dynamically monitor STING pathway activity in both primary and metastatic lesions and may play a significant role in future precision immunotherapy.

Circulating biomarkers are of considerable interest for clinical monitoring because of their minimally invasive nature and the advantage of repeat sampling. Serum levels of cytokines such as IFN-β and CXCL10 can reflect the degree of systemic STING activation (Yan et al., 2022). Similarly, the expression profile of ISGs in peripheral blood mononuclear cells (PBMCs) can be used to assess the pharmacodynamic effects of STING agonists. Furthermore, the methylation patterns and fragmentomic characteristics of circulating tumor DNA (ctDNA) can provide information about a tumor’s intrinsic immunogenicity. Exosomes, as important mediators of intercellular communication, carry cargo such as cGAMP or STING protein that may reflect STING signaling status within the tumor microenvironment (Wu et al., 2023). For instance, ENPP1, the primary hydrolase of extracellular cGAMP, shows expression levels correlated with tumor immune evasion, suggesting its potential as a novel circulating biomarker or therapeutic target (You et al., 2025). Research further reveals that tumor cell-derived exosomes can directly carry ENPP1 and hydrolyze cGAMP within immune cells, thereby inhibiting the cGAS–STING pathway and promoting immune escape, underscoring the importance of circulating exosomal ENPP1 as both a biomarker and therapeutic target (An et al., 2024). Additionally, tumor cell responses to metabolic stress may yield circulating markers. For example, under purine starvation stress, tumor cells assemble “purinosomes” and activate the cGAS–STING pathway; metabolites or DNA fragments released during this process could serve as novel circulating biomarkers for monitoring treatment response and tumor adaptation (Yu et al., 2025). The clinical utility of these circulating biomarkers still requires prospective validation through standardized procedures, but their potential for real-time monitoring and treatment adjustment is already emerging.

## 7 Future perspectives

### 7.1 Strategies for precision modulation

Given the complex dual role of the cGAS–STING pathway in tumor initiation and progression, future therapeutic strategies necessitate precise modulation of its activity. The overarching goal is to maximize anti-tumor immunity while concurrently minimizing potential pro-tumor risks. Spatiotemporally specific modulation represents a pivotal direction toward achieving this objective. Through targeted delivery systems, STING agonists can be efficiently enriched and released in a controlled manner within the tumor microenvironment, thereby effectively mitigating off-target toxicities associated with systemic immune activation (Samson and Ablasser, 2022; Lu et al., 2026). Furthermore, stimulus-responsive delivery systems leverage the unique physicochemical characteristics of the tumor microenvironment—such as weak acidity, hypoxia, or specific enzyme activities—to trigger precise drug release. This enables exact control over STING signaling activity in both temporal and spatial dimensions (Lu et al., 2026; Wang et al., 2024d). Such spatiotemporally precise modulation offers a viable path to resolve the therapeutic dilemma posed by the functional duality of the cGAS–STING pathway.

Precise regulation of signal strength constitutes another core strategy. Divergent levels of STING pathway activation often elicit markedly different biological outcomes: high-intensity activation typically induces tumor cell apoptosis and immunogenic cell death, whereas weak activation predominantly promotes cytokine production (Wang Q. et al., 2025). Consequently, fine-tuning the balance between pro-inflammatory and anti-inflammatory responses may be achieved through the rational design of agonist dosing regimens and exposure durations or by developing agonist variants with distinct intrinsic activities (Lu et al., 2026). Moreover, pulsatile dosing regimens, designed to mimic the transient yet potent activation of STING signaling during acute infections, may be more effective than continuous administration in preventing T-cell exhaustion and the establishment of immunosuppression (Dosta et al., 2025). Thus, the nuanced design of signal intensity and dynamic patterns introduces a new dimension for personalizing and optimizing immunotherapeutic interventions.

Selective modulation of downstream STING signaling pathways also presents significant clinical promise. Upon STING activation, two primary signaling axes are engaged: the TBK1–IRF3 pathway, which predominantly drives type I interferon production, and the NF-κB pathway, which mainly induces the expression of inflammatory cytokines. Therefore, developing STING agonists that preferentially activate IRF3 while avoiding NF-κB activation holds promise for enhancing anti-tumor immunity while limiting NF-κB-mediated pro-tumorigenic inflammation (Yue et al., 2025; Tanaka and Chen, 2012). Analogous strategies can be applied to cGAS regulation; for instance, designing allosteric modulators that subtly modulate its enzymatic activity could achieve functional selectivity rather than simply inducing complete activation or inhibition (Tanaka and Chen, 2012). This approach of finely tuned signal bias opens new avenues for enhancing anti-tumor efficacy while mitigating adverse effects.

Optimizing the timing and sequence of combination therapies is another critical determinant of improved efficacy. In preclinical studies, a regimen involving initial priming of innate immunity with a STING agonist to establish an initial anti-tumor response, followed by the introduction of immune checkpoint inhibitors to relieve T-cell suppression, has demonstrated superior synergistic effects (Jiang et al., 2020). Similarly, the combination of chemotherapy or radiotherapy with STING agonists requires meticulous scheduling: employing cytotoxic treatment first to induce tumor DNA damage and immunogenic cell death and subsequently administering a STING agonist to amplify and sustain the immune response, may yield better outcomes compared to concurrent administration (Sun K. et al., 2025). These complex issues related to therapeutic sequencing require systematic evaluation and validation in preclinical models to guide the design of more precise and effective combination immunotherapy strategies.

### 7.2 Optimization of combination therapeutic strategies

Optimizing combination therapeutic strategies is a central objective for overcoming current limitations and enhancing the clinical translational potential of STING agonists. Precision combination approaches based on tumor immune phenotyping hold significant promise for improving therapeutic efficacy. For immunologically “cold” tumors, characterized by scarce T-cell infiltration, combining STING agonists with other innate immune agonists (e.g., TLR agonists) or epigenetic modulators is often necessary to remodel the tumor immune microenvironment and facilitate conversion to a “hot” tumor phenotype (Sun K. et al., 2025). Conversely, for immunologically “hot” tumors with pre-existing T-cell infiltration, combining STING agonists with immune checkpoint inhibitors could directly amplify anti-tumor immune responses (Sun K. et al., 2025). In immunosuppressive tumors enriched with myeloid-derived suppressor cells or regulatory T cells, it is essential to combine STING agonists with agents targeting these suppressive populations to relieve immune blockade (Yue et al., 2025). Therefore, stratified intervention based on the heterogeneity of the tumor immune microenvironment represents a critical strategy for the precise and effective application of STING agonists.

The combination of multiple innate immune agonists is anticipated to yield potent synergistic effects. TLR agonists activate innate immune signaling pathways distinct from the STING pathway, and their combination can more comprehensively promote dendritic cell activation and antigen presentation (Cossu et al., 2023). RIG-I agonists activate the MAVS pathway upon recognition of cytosolic RNA, complementing the cGAS–STING pathway and synergistically enhancing type I interferon production (Qiao et al., 2020). NLRP3 agonists can induce pyroptosis, releasing a substantial amount of danger-associated molecular patterns, thereby amplifying the immune response triggered by STING activation (Mowat et al., 2025). This multi-pathway combination strategy aims to mimic the concurrent activation of multiple immune signaling pathways during pathogen infection, thereby eliciting more robust and durable anti-tumor immune responses.

The combination of STING agonists with metabolic interventions represents an emerging and promising research direction. Studies indicate that radiotherapy-induced increases in cholesterol synthesis can inhibit STING signaling, whereas statins can enhance the efficacy of STING agonists by inhibiting this metabolic pathway (Zhu L. et al., 2025). Glycolysis inhibitors can reduce lactate accumulation in the tumor microenvironment, alleviating its inhibitory effect on cGAS enzyme activity and consequently restoring and enhancing STING signaling (Xiang et al., 2023). Furthermore, modulating fatty acid oxidation can influence the metabolic reprogramming of immune cells such as T cells and dendritic cells, thereby synergizing with STING agonists to improve immune function. These metabolic interventions offer novel synergistic strategies to potentiate the efficacy of STING agonists.

The combination of STING agonists with epigenetic drugs also holds significant exploratory value. Many tumors suppress the STING pathway through epigenetic silencing mechanisms to achieve immune evasion (Sasaki et al., 2023). DNA methyltransferase inhibitors can reverse methylation of the STING promoter, thereby restoring its expression in tumor cells (Hu C. et al., 2021). HDAC inhibitors can enhance the transcriptional activity of interferon-stimulated genes by altering chromatin accessibility. EZH2 inhibitors, by removing the repressive H3K27me3 histone modification, can similarly upregulate STING expression (Marié et al., 2018). Therefore, combining epigenetic drugs with STING agonists holds promise for overcoming tumor-mediated suppression of STING signaling, fundamentally restoring pathway sensitivity and function.

Combination strategies that integrate STING agonists with conventional treatments such as chemotherapy, radiotherapy, and targeted therapies also require continuous optimization. While DNA damage-based therapies have a mechanistic basis for natural synergy with STING agonists, clinical application necessitates careful design regarding drug dosage and administration timing to avoid excessive toxicity (Lv et al., 2023). For instance, PARP inhibitors can enhance endogenous STING activation in tumors with DNA repair deficiencies, and their combination with STING agonists might induce synthetic lethality (Patterson-Forti et al., 2023; although this article has been retracted, the scientific concept remains informative). Similarly, inhibitors of ATM or ATR can enhance STING pathway activity by disrupting DNA damage repair signaling (Taniguchi et al., 2024). However, such combinations may increase toxicity to normal tissues, necessitating systematic preclinical assessment of their therapeutic window. Consequently, optimizing combination regimens should balance enhanced efficacy with safety control to promote the rational clinical application of STING agonists.

### 7.3 Development of novel agonists and delivery systems

The development of next-generation STING agonists is primarily focused on enhancing potency, improving pharmacokinetic profiles, achieving signaling selectivity, and overcoming inter-individual variability arising from genetic polymorphisms. Structure-based rational drug design, leveraging crystallographic information of STING protein–agonist complexes, enables the creation of small-molecule agonists with higher binding affinities and novel binding modes (Hines et al., 2023). Covalent agonists, by forming irreversible bonds with the STING protein, may achieve sustained pathway activation, although this approach necessitates heightened vigilance regarding the risk of excessive immune activation. Prodrug strategies involve introducing cleavable protecting groups onto agonist molecules to improve their stability and cell membrane permeability, ensuring that the active compound is released only upon reaching its target (Zhou et al., 2023). These strategies are complementary and collectively drive the development of STING agonists toward safer and more effective therapeutic options.

The development of orally available STING agonists is critical for enhancing medication convenience and patient compliance. MSA-2, reported as the first oral non-nucleotide STING agonist, provided pivotal proof of concept for this direction (Pan et al., 2020). However, its clinical efficacy still requires further validation. Prodrug design is a common strategy to improve oral bioavailability, but it must carefully consider factors such as drug stability in the gastrointestinal tract and hepatic first-pass metabolism. Furthermore, the potential influence of the gut microbiota on the metabolism and activity of oral agonists warrants attention. Moving forward, the development of STING agonists that combine favorable oral activity with a controllable safety profile remains a major area of innovation in the field.

Developing agonists that target specific STING subtypes and genetic variants holds significant clinical importance. Designing highly selective agonists for common human STING haplotypes, such as R232 and HAQ, could effectively overcome the inter-individual differences in drug response caused by genetic polymorphisms (Froechlich et al., 2023; Koide et al., 2025). Additionally, STING splice variants specifically expressed in tumors may represent highly selective therapeutic targets; however, the function of these variants and their potential as drug targets require in-depth investigation. Achieving precise regulation of distinct STING variants could pave the way for personalized immunotherapy.

Innovations in combination delivery systems can concurrently address multiple challenges in drug delivery and combination therapy. Multi-drug co-delivery nanoparticles, loaded simultaneously with a STING agonist, immune checkpoint inhibitors, and chemotherapeutic agents, enable the synchronized delivery of multimodal treatments for enhanced synergistic effects (Huang et al., 2025). Temporal release systems, designed with materials featuring layered degradation properties or responsiveness to different stimuli, can release different drugs in a pre-programmed sequence, thereby optimizing the timing of combination therapies (Wells et al., 2019). Targeted delivery systems, through the conjugation of specific antibodies or ligands on their surface, can achieve selective delivery to tumor cells, specific immune cells, or stromal cells. This allows for precise modulation of STING activity in particular cell populations, significantly reducing systemic toxicity while enhancing therapeutic efficacy (Peng et al., 2025; Chen L. et al., 2021). The synergistic optimization of these delivery systems provides crucial tools for realizing precise and controllable immune combination therapy.

Live-cell-based delivery strategies utilize engineered cells as “micro-pharmacies” and targeting vehicles for STING agonists. For instance, engineered CAR-T cells can be designed to release cGAMP in a regulated manner within the tumor microenvironment, thereby boosting their own anti-tumor activity (Chen L. et al., 2021). Certain tumor-tropic bacteria, such as attenuated *Salmonella*, can serve as vehicles to deliver STING agonists specifically to hypoxic tumor regions. Red blood cells or platelets, as natural carriers, offer excellent biocompatibility and prolonged circulation times (Wu et al., 2024). While these innovative cell-based delivery strategies demonstrate significant potential, finding an optimal balance between efficacy and safety is paramount, particularly in avoiding severe toxicities such as cytokine release syndrome. Managing the systemic risks associated with enhanced immune activation will be a critical test for the clinical translation of these approaches.

### 7.4 Personalized therapeutic strategies

The realization of personalized therapies targeting the cGAS–STING pathway hinges on the integration of multidimensional patient data—including tumor genomic features, immune microenvironment status, and host genetic background—to formulate more precise treatment strategies. The most direct approach currently involves screening patients most likely to benefit based on tumor genomic characteristics. For instance, tumors with high microsatellite instability typically exhibit a high mutational burden and immunogenicity, often accompanied by high STING pathway expression, making them a potentially suitable population for STING agonist therapy (Ying et al., 2024). Furthermore, tumors harboring mutations in DNA damage repair genes (such as BRCA1/2 deficiency) are more sensitive to STING signaling induced by endogenous DNA damage and may also derive greater benefit from STING agonist treatment (Xu et al., 2025; Yadav et al., 2025). Concurrently, the polymorphic status of the patient’s own STING gene must be carefully considered when selecting agonists and adjusting dosages (Wang J. et al., 2022). Therefore, a comprehensive characterization of the tumor genome constitutes a fundamental step in personalizing cGAS–STING pathway-targeted therapies.

The characteristics of the immune microenvironment provide crucial guidance for selecting combination treatment strategies. For instance, “hot” tumors with abundant T-cell infiltration may be suitable for STING agonist monotherapy or combination with immune checkpoint inhibitors. In contrast, immunosuppressive tumors enriched with myeloid-derived suppressor cells or regulatory T cells may require combination with agents targeting these populations to relieve immunosuppression (Yue et al., 2025). For tumors with dysfunctional dendritic cells, combining STING agonists with dendritic cell activators or cancer vaccines might be necessary to enhance antigen presentation. These complex immune phenotypes can be accurately assessed using advanced techniques such as multiplex immunohistochemistry, mass cytometry, or single-cell sequencing. Hence, precise dissection of the immune microenvironment is instrumental in optimizing combination regimens and enhancing therapeutic efficacy.

Dynamic monitoring during treatment is equally critical for timely adjustment of therapeutic strategies. Circulating biomarkers, such as serum levels of IFN-β and CXCL10, can reflect the real-time activation status of the STING pathway, providing reference points for dose modification. Changes in the proportions and numbers of peripheral blood immune cell subsets (e.g., activated CD8^+^ T cells) can be used to evaluate the establishment of adaptive immune responses. The clearance kinetics of circulating tumor DNA can reflect the drug’s anti-tumor effect, aiding decisions on whether to continue the current therapy or switch regimens early. Additionally, imaging assessments can evaluate changes in tumor burden, guiding the optimal timing for local interventions such as radiotherapy or surgery. Therefore, establishing a multi-dimensional dynamic monitoring system is essential for closing the loop in personalized treatment management.

Mechanism-based functional assays hold promise for providing more precise guidance for decision-making in personalized therapy. The use of patient-derived tumor organoids or humanized mouse models allows for *ex vivo* or *in vivo* assessment of tumor sensitivity to STING agonists, potentially enabling prediction of clinical efficacy (Chen et al., 2024; Liu L. C. et al., 2022). These models can, to some extent, retain features of the original tumor microenvironment, offering functional data that more closely mirrors *in vivo* reality than genomic testing alone. However, standardizing these methods, reducing costs, and improving their clinical accessibility remain significant challenges. Consequently, advancing the clinical translation of functional assay technologies represents a crucial direction for the precise personalization of cGAS–STING pathway therapies.

In summary, achieving personalized treatment targeting the cGAS–STING pathway requires not only the integrated analysis of multi-dimensional data but also the development and standardization of dynamic monitoring tools and functional testing models. With continuous advances in precision medicine technologies, constructing patient-centric personalized treatment pathways holds significant promise for substantially improving the clinical benefits of cGAS–STING-targeted therapies.

## 8 Conclusion

The cGAS–STING pathway, consistent with its role in connecting innate and adaptive immunity, plays a complex and central role in tumor immunity. This review systematically elucidates the molecular mechanisms of this pathway, its dual role in cancer, the mechanisms underlying its dysregulation, and the research progress in targeted therapeutic strategies, leading to the following main conclusions.

First, the activation mechanism of the cGAS–STING pathway is highly sophisticated. cGAS catalyzes the synthesis of the second messenger cGAMP upon recognition of cytosolic DNA, which subsequently activates the endoplasmic reticulum-anchored protein STING. Activated STING undergoes a conformational change and translocates from the endoplasmic reticulum to the Golgi apparatus, where it recruits and activates TBK1 via its C-terminal tail. TBK1 then phosphorylates IRF3, ultimately inducing the production of type I interferons and pro-inflammatory cytokines. This process is finely tuned by a variety of post-translational modifications, including ubiquitination, phosphorylation, SUMOylation, and palmitoylation, ensuring a timely and measured immune response.

Second, the pathway’s role in cancer is highly context-dependent, capable of exerting opposing effects depending on the stage and setting of tumor progression. On one hand, by sensing tumor-derived DNA, it activates a type I interferon response, promoting dendritic cell maturation and CD8^+^ T-cell infiltration, thereby exerting potent anti-tumor immune surveillance. Clinical studies indicate that high expression of cGAS or STING positively correlates with a favorable prognosis in patients with various cancers. On the other hand, in advanced stages of tumor progression, chronic and persistent activation predominantly drives inflammation via the NF-κB pathway, shaping an immunosuppressive microenvironment. This fosters the infiltration of myeloid-derived suppressor cells and regulatory T cells, upregulates co-inhibitory molecules such as PD-L1, and can even directly confer tumor cells with enhanced stemness, survival capacity, and therapy resistance. This functional duality depends on a delicate balance of factors, including signal dynamics, activation intensity, cell-type specificity, and the characteristics of the tumor microenvironment.

Third, tumor cells have evolved multi-layered mechanisms to evade immune surveillance mediated by the cGAS–STING pathway. At the epigenetic level, pathway silencing is achieved through promoter methylation of cGAS and STING genes and aberrant histone modifications. At the protein level, pathway activity is modulated by post-translational modifications such as ubiquitination (e.g., by TRIM6 and TRIM27), phosphorylation (e.g., AKT-mediated phosphorylation of cGAS at Ser291), and SUMOylation. At the level of autophagy, tumor cells hijack autophagic machinery to clear cytosolic DNA and activated STING. Furthermore, within the tumor microenvironment, factors such as hypoxia, metabolic reprogramming (e.g., increased cholesterol synthesis), acidosis, and cancer-associated fibroblasts profoundly influence cGAS–STING pathway activity.

Fourth, significant progress has been made in therapeutic strategies targeting the cGAS–STING pathway, yet challenges persist. The development of STING agonists has expanded from cyclic dinucleotides (e.g., ADU-S100 and MK-1454) to non-nucleotide small molecules (e.g., diABZI and MSA-2). Combination strategies involving immune checkpoint inhibitors, chemotherapy, radiotherapy, and targeted drugs have shown immense potential in preclinical studies. Nanodelivery systems (including liposomes, polymeric nanoparticles, inorganic nanomaterials, metal-organic frameworks, and biomimetic systems) hold promise for overcoming current therapeutic limitations by improving the pharmacokinetic properties and tumor targeting of agonists. However, preliminary clinical trial results have been less impressive than anticipated, with major challenges including: the exhausted immune state in late-stage patients, inherent pharmacokinetic deficiencies of CDNs, multiple resistance mechanisms in tumor cells, STING genetic polymorphisms, and the heterogeneity of the tumor microenvironment.

Fifth, future directions are focused on precision, personalization, and intelligent design. In terms of precision modulation, achieving spatiotemporally specific delivery, optimization of signal intensity, and selective regulation of downstream pathways is crucial. For combination therapies, strategies should be developed based on tumor immune phenotyping, with exploration of synergistic combinations with metabolic interventions, epigenetic drugs, and other innate immune agonists. Regarding delivery systems, future efforts should focus on developing orally bioavailable agonists, variant-specific STING agonists, and multifunctional combination delivery systems. For personalized treatment, it is necessary to integrate tumor genomic features (e.g., MSI status and DDR gene mutations), immune microenvironment characteristics, and STING genetic polymorphisms to identify optimal patient populations and guide treatment adaptation through dynamic monitoring of circulating biomarkers.

In summary, the cGAS–STING pathway represents a crucial target for cancer immunotherapy. Its complexity and duality demand systematic and in-depth exploration across fundamental research, drug development, and clinical translation. With a deeper understanding of the spatiotemporal regulatory mechanisms of this pathway, and the continuous emergence of novel agonists, smart delivery systems, and precision combination strategies, therapies targeting the cGAS–STING pathway are poised to become a significant component of cancer immunotherapy, offering clinical benefits to a greater number of patients.
